# Role of PCNA and RFC in promoting Mus81-complex activity

**DOI:** 10.1186/s12915-017-0429-8

**Published:** 2017-10-02

**Authors:** Alexandra Sisakova, Veronika Altmannova, Marek Sebesta, Lumir Krejci

**Affiliations:** 1 Department of Biology, Masaryk University, Kamenice 5/A7, CZ-62500 Brno, Czech Republic; 2 National Centre for Biomolecular Research, Masaryk University, Kamenice 5/A4, CZ-62500 Brno, Czech Republic; 3 International Clinical Research Center, Center for Biomolecular and Cellular Engineering, St. Anne’s University Hospital Brno, Pekarska 53, CZ-656 91 Brno, Czech Republic; 4 Present address: Sir William Dunn School of Pathology, University of Oxford, South Parks Road, Oxford, OX1 3RE UK

**Keywords:** Replication factor C, Proliferating cell nuclear antigen, Mus81 complex, Replication, Recombination

## Abstract

**Background:**

Proper DNA replication is essential for faithful transmission of the genome. However, replication stress has serious impact on the integrity of the cell, leading to stalling or collapse of replication forks, and has been determined as a driving force of carcinogenesis. Mus81-Mms4 complex is a structure-specific endonuclease previously shown to be involved in processing of aberrant replication intermediates and promotes POLD3-dependent DNA synthesis via break-induced replication. However, how replication components might be involved in this process is not known.

**Results:**

Herein, we show the interaction and robust stimulation of Mus81-Mms4 nuclease activity by heteropentameric replication factor C (RFC) complex, the processivity factor of replicative DNA polymerases that is responsible for loading of proliferating cell nuclear antigen (PCNA) during DNA replication and repair. This stimulation is enhanced by RFC-dependent ATP hydrolysis and by PCNA loading on the DNA. Moreover, this stimulation is not specific to Rfc1, the largest of subunit of this complex, thus indicating that alternative clamp loaders may also play a role in the stimulation. We also observed a targeting of Mus81 by RFC to the nick-containing DNA substrate and we provide further evidence that indicates cooperation between Mus81 and the RFC complex in the repair of DNA lesions generated by various DNA-damaging agents.

**Conclusions:**

Identification of new interacting partners and modulators of Mus81-Mms4 nuclease, RFC, and PCNA imply the cooperation of these factors in resolution of stalled replication forks and branched DNA structures emanating from the restarted replication forks under conditions of replication stress.

**Electronic supplementary material:**

The online version of this article (doi:10.1186/s12915-017-0429-8) contains supplementary material, which is available to authorized users.

## Background

During DNA replication, endogenous and exogenous factors can cause replication forks (RFs) to stall and/or collapse, a condition commonly known as replication stress [1, 2]. Common fragile sites are particularly unstable under replication stress; they are DNA sequences, scattered throughout the genome, that are intrinsically difficult to replicate due to their inherent propensity to form secondary structures, causing RF stalling. These events may be associated with occurrence of ssDNA gaps and/or DNA breaks [2, 3]. Correct progression and termination of DNA replication is essential to maintain genome integrity of the cell [4], since genomic instability resulting from replication stress has been recognized as a driver in carcinogenesis [5]. Cells have evolved multiple pathways to restart stalled RFs, with one of the mechanisms involved being homologous recombination through stabilization of RFs and invasion into homologous sequences found within the sister chromatid, resulting in a transient formation of a sister-chromatid junction [6]. However, prolonged accumulation of these intermediate structures is toxic for the cell. Therefore, homologous recombination requires the action of the Sgs1-Top3-Rmi1 complex, which catalyzes dissolution of DNA intermediates, yielding exclusively non-crossover products and thereby suppressing crossover formation [7]. Alternatively, a battery of structure-specific endonucleases (Yen1, Slx1-Slx4, Mus81-Mms4) can cleave the joint DNA structures [8].

The Mus81-Mms4 complex (henceforth named the Mus81 complex) is a highly conserved, structure-specific endonuclease. It is a member of the XPF family of proteins that plays a crucial role in maintaining genome integrity in various DNA processing pathways [9]. This complex is implicated in resolution of recombination intermediates, in inter-strand DNA cross-link repair, and in the recovery of stalled or blocked RFs [8]. In vitro, the Mus81 complex shows preference for substrates mimicking RFs, namely 3′ flap structures, nicked Holliday junctions, and D-loop structures (mimicking sister-chromatid junctions occurring in vivo) [6]. Recent studies have demonstrated that the activity of the Mus81 complex is regulated during the cell cycle by Cdk1 and Cdc5 kinases, as well as via protein-mediated interactions [10–12]. Interestingly, a recent study in yeast revealed another cell cycle kinase, Cdc7-Dbf4, which binds and phosphorylates the Mus81 complex in mitosis, and in association with Cdc5 is required for full Mus81 complex activation [13]. The activation of the complex occurs by phosphorylation of the Mms4 subunit at G2/M transition to assure the resolution of joint DNA structures that persist after the bulk DNA synthesis has occurred but prior to chromosome segregation. At the same time, post-replicative activation of the Mus81 complex limits potentially undesirable cleavage of normal RFs during S phase that may lead to double-strand break (DSB) formation [10, 14]. Accordingly, WEE1 in human cells was shown to restrain CDK1-mediated assembly of MUS81-SLX4 in S phase, which is required for efficient substrate targeting [15]. In addition, several factors involved in DNA replication and repair (in yeast: Rad27, Rad54, Srs2, Esc2, Rad52; in mammalian cells: FEN1, BLM, RAD54, RAD52) have been shown to stimulate nuclease activity of the Mus81 complex [16–20]. All these data clearly indicate the need for tight control and regulation of Mus81 complex activity.

Human MUS81-EME1 promotes replication restart by converting collapsed RFs into DNA DSBs upon replication inhibition [21, 22]. Moreover, a recent study revealed that the human MUS81 complex plays an important role in common fragile site expression, occurring in the early mitotic phase of the cell cycle. Under these circumstances, activity of the MUS81 complex promotes POLD3 (human orthologue of yeast Pol32)-dependent DNA synthesis via the break-induced replication (BIR) mechanism [23]. Fittingly, yeast cells defective in either subunit of the nuclease complex exhibit sensitivity to agents causing RF stalling such as camptothecin (CPT), hydroxyurea (HU), and methyl methanesulfonate (MMS) [24]. Furthermore, Mus81 plays an essential role in limiting mutagenicity during BIR, which can be highly inaccurate and prone to switching templates [25], by cleavage of unstable D-loop structure and thus stabilizing the fork structure [26]. An active role of the MUS81 complex in timely replication has also been suggested, as MUS81-deficient cells fail to recover from replication inhibitors, and viability of MUS81-defiecient cells depends on XPF nuclease [27]. Yeast *MUS81* and *MMS4* have been shown to genetically interact with several DNA replication factors. In *S. pombe*, the Mus81 complex collaborates with Rad2, a FEN1 and Rad27 ortholog, during Okazaki fragment maturation [28], further corroborating the notion that the Mus81 complex is required for completion of DNA replication.

Proliferating cell nuclear antigen (PCNA) is the key component of replication machinery. It functions as a trimeric complex that encircles DNA, serving as a processivity factor of DNA polymerases, historically known as the sliding clamp. For its proper activity, it must be actively loaded onto DNA. This process requires a clamp loader, replication factor C (RFC), which loads PCNA onto primer-template junctions in an ATP-dependent manner [29, 30]. RFC is a heteropentameric complex composed of a large Rfc1 subunit (94.9 kDa) and four small subunits – Rfc2 (39.7 kDa), Rfc3 (38.2 kDa), Rfc4 (36 kDa), and Rfc5 (40 kDa). All five subunits contain highly conserved regions known as RFC boxes (II–VIII), which are essential for cell viability [31]. Importantly, certain mutations in *RFC2*, *RFC4*, and *RFC5* exhibit sensitivity to HU and MMS [32], indicating that active PCNA loading may play a role in DNA repair. There are three paralogs of the *RFC1* gene present in eukaryotes, *CTF18*, *RAD24*, and *ELG1*, which form alternative, RFC-like complexes (RLCs) with the smaller subunits (Rfc2–5) [33–36].

In this study, we investigated the possibility that the RFC and Mus81 complexes cooperate under conditions of replication stress in resolution of stalled RFs and branched DNA structures emanating from the restarted RFs. We first established that these two complexes interact in vitro and in vivo. Next, we studied the functional consequence of the newly described interaction. We found that the RFC complex robustly stimulates the endonuclease activity of the Mus81 complex in vitro. Moreover, this stimulation is further enhanced by ATPase activity of the RFC complex, and also by the PCNA loading on the DNA substrate. We also demonstrate that this stimulation is not specific to the Rfc1 subunit, as other RLCs (Elg1-RLC) can stimulate the activity of the Mus81 complex. We further corroborated our in vitro observation by analyzing the genetic interaction between a diverse set of mutant RFC alleles and *MUS81.* We found an epistatic relationship between these two factors in presence of MMS, indicating cooperation of RFC and Mus81 complex in repair of lesions under this condition. Interestingly, the sensitivity of RFC mutants to cis-platin (CisPT) and zeocin (ZEO) was suppressed in *∆mus81* background, signifying toxic intermediates might be generated by the Mus81 complex in this mutant. Our data point to the possibility that, under particular instances of replicative stress, the activity of the Mus81 complex can be promoted by a component of a replisome, the RFC complex.

## Results

### PCNA interaction with the Mus81 complex and its effect on the Mus81 complex nuclease activity

**Fig. 1:**
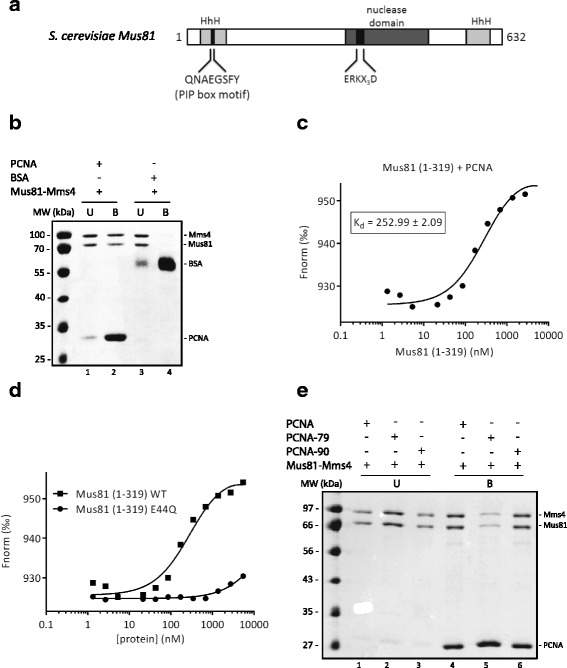
Interaction of the Mus81-Mms4 complex with PCNA. **a** Schematic representation of Mus81 protein with marked PIP box motif. **b** Purified recombinant Mus81-Mms4 (5 μg) was mixed either with PCNA or BSA covalently bound to Affi-beads in Tris buffer containing 100 mM KCl. After 30 min incubation at 4 °C the supernatant was removed and the beads were washed twice with Tris buffer containing 150 mM KCl. The unbound (U) and bound (B) fractions were then analyzed on 12% SDS gel. **c** Microscale thermophoresis measurement of interaction between PCNA and Mus81 (1–319). Fluorescently labeled PCNA (22 nM) was mixed with increasing concentrations (1.3–5500 nM) of Mus81 (1–319). The binding affinity of PCNA towards Mus81 (1–319) was quantified by MO.Affinity Analysis software. **d** Microscale thermophoresis measurement of the interaction between PCNA and Mus81 (1–319) wild-type and PIP-box mutant. Fluorescently labeled PCNA (22 nM) was mixed with increasing concentrations (1.3–5500 nM) of Mus81 (1–319) wild-type or E44Q mutant. The binding affinity of PCNA towards Mus81 variants was quantified by MO.Affinity Analysis software. **e** Purified recombinant Mus81-Mms4 (2.5 μg) was mixed with PCNA, PCNA-79, or PCNA-90 covalently bound to Affi-beads in Tris buffer containing 150 mM KCl. After 30 min incubation at 11 °C, the supernatant was removed and the beads were washed twice with Tris buffer containing 150 mM KCl. The reactions were analyzed as shown in **b**

The Mus81 complex has been suggested to play a role in processing stalled RFs [37]. We therefore wanted to test whether components of replication machinery can interact with the Mus81 complex and affect its enzymatic activity. The sequence analysis of Mus81 protein revealed the presence of a non-canonical PCNA interaction motif (PIP box) at its N-terminus (Fig. 1a). To verify the possible interaction between PCNA and the Mus81 complex, we generated PCNA Affi-beads to pull-down the purified Mus81 complex. Using this approach, we observed direct physical interaction between PCNA and the Mus81 complex (Fig. 1b, lanes 1 and 2). In a control experiment, we did not observe the Mus81 complex retained on bovine serum albumin (BSA)-conjugated Affi-beads (Fig. 1b, lanes 3 and 4), pointing to the specific interaction between PCNA and the Mus81 complex. We also employed microscale thermophoresis (MST) analysis (Fig. 1c) and observed direct binding between PCNA and the N-terminal fragment of Mus81 (1–319) with apparent K_d_ = 252.99 ± 2.09 nM. To determine whether this interaction utilizes the PIP box motif, we performed a pull-down experiment in the presence or absence of the short peptides containing wild-type (pFF) or mutated (pAA) PIP box motifs, respectively. We observed that the pFF peptide, unlike the pAA peptide, inhibited binding of the Mus81 complex to PCNA (Additional file 1: Figure S1B). The importance of PIP box is also supported by the MST experiment with the N-terminal fragment of Mus81-containing (1–319) mutation in the PIP box (Mus81-E44Q). Importantly, this mutant binds PCNA with significantly lower affinity (K_d_ = 20498.6 ± 0.49 nM) (Fig. 1d). To determine which region of PCNA is responsible for Mus81 binding, we performed a pull-down experiment using the Mus81 complex and a set of PCNA mutants containing mutations within the interdomain connector loop (IDCL) and C-terminus, known to be defective in protein–protein interactions [38, 39]. The IDCL domain mutant *pcna-79* (IL 126, 128 AA) and the C-terminal mutant *pcna-90* (PK 252, 253 AA), respectively, were immobilized on Affi-beads to pull-down the Mus81 complex. As shown in Fig. 1e, the interaction between the Mus81 complex and *pcna-79* is significantly decreased compared to the wild-type PCNA and *pcna-90*, suggesting that the interaction is mediated via the IDCL domain on PCNA.

**Fig. 2:**
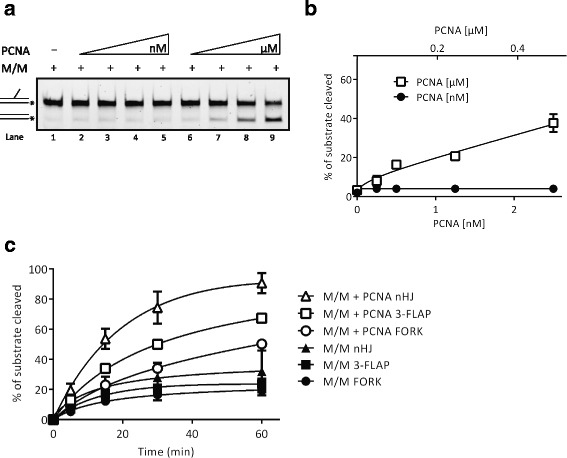
PCNA-dependent enhancement of Mus81-Mms4 activity. **a** Reaction mixtures containing DNA substrate (5 nM), Mus81-Mms4 (0.25 nM), and increasing amounts of PCNA (0.25, 0.5, 1.25, and 2.5 nM, or 0.05, 0.1, 0.25, and 0.5 μM) were incubated at 37 °C for 20 min and then analyzed. **b** Quantification of data from three independent experiments. **c** Effect of PCNA on Mus81-Mms4 nuclease activity on various DNA substrates. Mus81-Mms4 (0.2 nM) was incubated in the absence or presence of PCNA (0.5 μM) at 37 °C for the indicated time. The data from three independent experiments were quantified. *Raw data provided in Additional file 2

The interaction between PCNA and the Mus81 complex prompted us to determine the functional consequences of this interaction on the nuclease activity of the Mus81 complex. To evaluate this effect, we used a 3′ flap DNA substrate, a standard substrate for the Mus81 complex. A sub-stoichiometric amount of the Mus81 complex (0.25 nM) was used in the assay, corresponding to the concentration of the enzyme cleaving approximately 5% of DNA substrate (Fig. 2a, lane 1; Additional file 2). Addition of increasing amounts of PCNA resulted in stimulation of nuclease activity of the Mus81 complex, albeit only at very high PCNA concentrations (Fig. 2a, lanes 6–9; Additional file 2), thereby indicating the role of PCNA in recruiting the Mus81 complex. Since the Mus81 complex was shown to cleave other replication and recombination intermediates, we also tested the effect of PCNA on cleavage of fork and nicked Holliday junction structures. The time-course experiments revealed that PCNA promotes the Mus81 complex nuclease activity on all tested substrates (Fig. 2c, Additional file 1: Figure S1C, D and E, and Additional file 2). The effectiveness of this stimulation was comparable on all DNA substrates, even though the cleavage of nicked Holliday junction structures showed the highest efficiency in the presence of PCNA in comparison to the reaction with the Mus81 complex alone (in time point 60 min).

Taken together, PCNA interacts specifically with the Mus81 subunit of the Mus81 complex, and this interaction is mediated by the PIP box motif and promotes the nuclease activity of the Mus81 complex on the variety of DNA substrates.

### Mus81 complex interacts with RFC complex

**Fig. 3:**
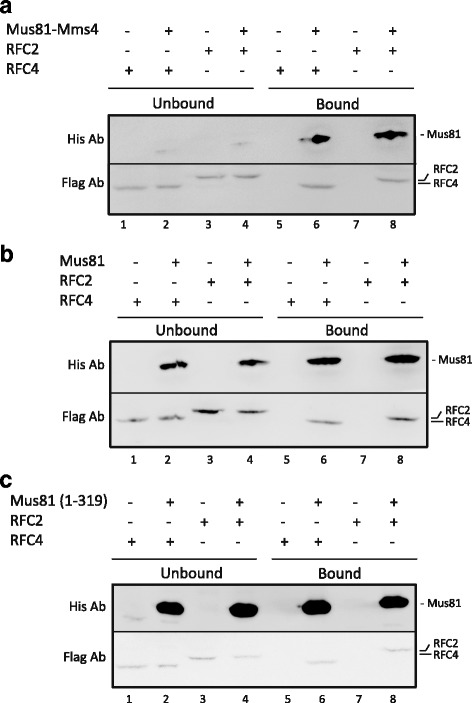
Physical interaction of RFC2 and RFC4 with Mus81-Mms4 or Mus81. **a** Purified recombinant Mus81-Mms4 containing His_6_-tag on Mms4 was mixed with yeast cell extracts (150 μL) containing Flag-tagged RFC2 or RFC4 and captured on Ni-NTA (lanes 2, 4, 6, and 8). After 30 min, the supernatant was removed, the beads washed, and bound proteins eluted. The fraction containing unbound proteins (Unbound) and the SDS eluates (Bound) were analyzed on SDS-PAGE followed by immunoblotting using anti-Flag and anti-His antibodies. As a control, yeast cell extracts containing either Flag-tagged RFC2 or RFC4 were mixed with Ni-NTA beads in the absence of the Mus81 complex (lanes 1, 3, 5, and 7). The positions of individual proteins are marked on the side of the gel. **b** Interaction of RFC2 or RFC4 with Mus81 subunit. The assay was performed as shown in (a). **c** Interaction of RFC2 or RFC4 with Mus81 (1–319) fragment. The assay was performed as shown in (a)

PCNA is loaded onto DNA by the RFC complex; we therefore reasoned that this complex may also affect the activity of the Mus81 complex. To test this hypothesis, we first analyzed whether the Mus81 complex interacts with the RFC complex. Initially, we performed an in vivo pull-down assay with tagged versions of RFC2 and RFC4. Yeast cells containing Flag-tagged RFC2 and RFC4 subunits, respectively, were lysed and the cell extracts were mixed with the Mus81 complex containing a His_6_-tag. The mixture was then incubated with Ni-NTA beads, bound proteins were eluted, and the elution and supernatant fractions analyzed by SDS-PAGE, followed by immunoblotting using specific antibodies against His_6_ and Flag tags, respectively (Fig. 3). This assay revealed significant interaction between the Mus81 complex and the RFC complex (Fig. 3a). To determine the subunit of the Mus81 complex responsible for the interaction, we repeated the assay with Mus81 protein alone. As seen in Fig. 3b, this subunit is able to interact with RFC2 and RFC4. To narrow down the interaction domain, we also tested the N-terminal fragment of Mus81 (Mus81 1–319). Using this polypeptide we were able to pull-down both RFC subunits, indicating that the RFC binding domain of Mus81 protein is located within the first 319 amino acids (Fig. 3c). Taken together, these data show that the Mus81 complex associates with the RFC complex and that this interaction is mediated by the N-terminal part of the Mus81 protein.

### RFC stimulates Mus81-Mms4 and Rad27 nuclease activities

**Fig. 4:**
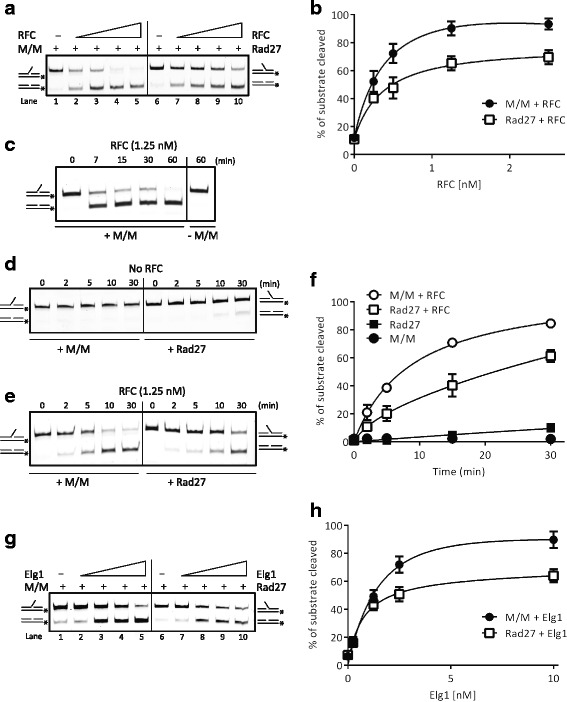
RFC and Elg1 stimulate the Mus81 complex and Rad27 nuclease activities. **a** Reaction mixtures containing DNA substrate (5 nM) and the Mus81 complex (M/M) (0.25 nM, lanes 1–5) or Rad27 (0.25 nM, lanes 6–10) were incubated with increasing amounts of the RFC complex (0.25, 0.5, 1.25, and 2.5 nM) at 37 °C for 20 min and then analyzed. **b** Quantification of data from three independent experiments. **c** RFC alone does not cleave the DNA substrate. Time-course enhancement of the Mus81 complex activity by RFC. The Mus81 complex (0.25 nM) was incubated with the 3′ flap DNA substrate (5 nM) at 37 °C for 60 min in the presence of RFC (1.25 nM). Aliquots of the reactions were taken at the indicated times and analyzed. Last lane indicates the effect of RFC complex on the DNA substrate in the absence of the Mus81 complex. **d, e** Time course enhancement of the Mus81 complex and Rad27 cleavage by RFC. The Mus81 complex (M/M, 0.25 nM) and Rad27 (0.25 nM) were incubated with the 3′ flap or 5′ flap DNA substrates (5 nM) at 37 °C for 30 min (**d**) in the absence or (**e**) in the presence of RFC (1.25 nM). Aliquots of the reactions were taken at the indicated times and analyzed. **f** Quantification of data in (d) and (e) from three independent experiments. **g** Reaction mixtures containing DNA substrate (5 nM), Mus81-Mms4 (0.25 nM, lanes 1–5) or Rad27 (0.25 nM, lanes 6–10) and increasing amounts of Elg1-RFC complex (0.25, 1.25, 2.5, and 10 nM) were incubated at 37 °C for 20 min and then analyzed. **h** Quantification of data in (g) from three independent experiments. *Raw data provided in Additional file 2

The physical interaction between the Mus81 and the RFC complexes prompted us to test whether RFC affects the nuclease activity of the Mus81 complex. Since human FEN1 has been previously reported to be robustly stimulated by RFC complex [40], we decided to compare the effect of RFC on activities of the Mus81 complex as well as Rad27, the yeast FEN1 orthologue. To monitor the effect, we used 3′ flap and 5′ flap DNA substrates, mimicking standard substrates for Mus81-Mms4 and Rad27, respectively. Suboptimal concentrations of the Mus81 complex and Rad27 (0.25 nM) were used in the assay, which correspond to the concentration of the enzyme cleaving approximately 10% of the respective DNA substrate (Fig. 4a, lanes 1 and 6). The addition of increasing amounts of RFC (0.25, 0.5, 1.25, or 2.5 nM) to the reaction mixture robustly stimulated the activity of both nucleases (Fig. 4a and b, lanes 2–5, 7–10; Additional file 2). Intriguingly, the RFC complex reproducibly stimulated the activity of the Mus81 complex up to 8-fold, compared to a 6-fold stimulation of the activity of Rad27. This stimulation was not due to a contaminating nuclease activity present in the purified RFC complex as its addition alone had no effect on corresponding DNA substrate (Fig. 4c). Next, we examined the rate of the reaction using a time-course experiment (Fig. 4d, e and f; Additional file 2). In the absence of the RFC complex, no cleaved product was detectable even after 30 min of the addition of the Mus81 complex, and only 9% of the product was detectable if Rad27 was used (Fig. 4d). However, in the presence of the RFC complex, we observed significant acceleration of substrate cleavage by both the Mus81 complex and Rad27, respectively (Fig. 4e). Specifically, after 5 min, we detected 20% cleavage of the substrate in the reaction containing Rad27 and RFC in contrast to 40% cleavage in the presence of the Mus81 complex. These results indicate that the RFC complex dramatically stimulates the nuclease activity of both the Mus81 complex as well as Rad27. However, there is a significantly higher stimulation of the Mus81 complex, compared to that of Rad27, by the RFC complex.

To determine whether this stimulation is species-specific, we performed an experiment with human MUS81-EME1 complex. As shown in Additional file 3: Figure S2A, the yeast RFC complex was also able to stimulate the activity of the human MUS81 complex, indicating that the stimulation mechanism is general and not species-specific.

Similarly as for PCNA, we also analyzed the effect of RFC complex on Mus81 nuclease activity on fork structures and nicked Holliday junctions. Expectedly, we observed substantial stimulation of the Mus81 complex enzymatic activity on both substrates in the level comparable to 3′ flap DNA (Additional file 3: Figure S2B–G; Additional file 2).

### The Mus81 complex and Rad27 are stimulated by Elg1-RLC

To date, three alternative RFC-like complexes have been described, the Ctf18-RLC, Rad24-RLC, and Elg1-RLC, respectively. To determine whether the stimulation of the Mus81 complex activity by RFC is specific to the canonical one, we decided to test the effect of an alternative RFC-like complex in which the Rfc1 subunit is replaced by the Elg1 protein. To test the ability of Elg1-RLC to stimulate the Mus81 nuclease activity, we performed an experiment similar to that described above (Fig. 4). The Mus81 complex and Rad27 (both at 0.25 nM) were incubated with increasing amounts of Elg1-RLC (0.25–10 nM). Similarly as with the RFC complex, we observed robust stimulation of both the Mus81 complex and Rad27 by Elg1-RLC complex (Fig. 4g, h; Additional file 2). Specifically, at 2.5 nM Elg1-RLC we observed 7- and 13-fold stimulation of the nuclease activity of Rad27 and the Mus81 complex, respectively.

### Effect of both RFC and PCNA on activity of the Mus81 complex

Since RFC and PCNA form a functional complex during DNA replication and repair, we also investigated the effect of the simultaneous presence of PCNA and RFC on the enzymatic activity of the Mus81 complex. We found that the addition of both RFC (0.25–2.5 nM) and PCNA in the reaction did not increase the quantity of cleaved product compared to the reactions with RFC alone (Additional file 1; Additional file 4: Figure S3A, B).

**Fig. 5:**
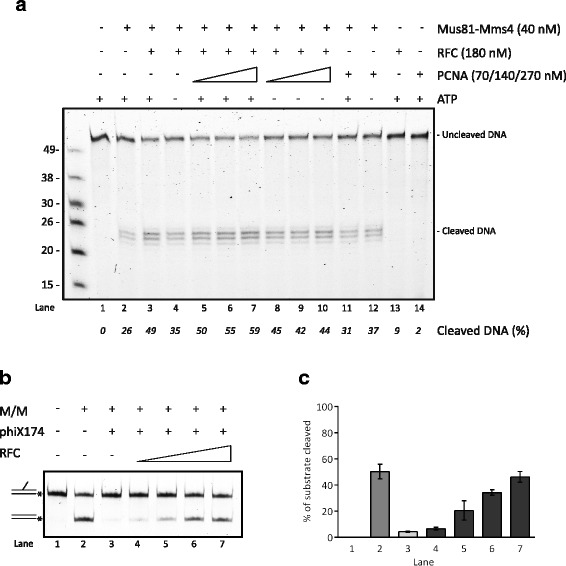
The influence of PCNA loading and RFC targeting on nuclease activity of the Mus81 complex. **a** Effect of RFC-mediated PCNA loading on stimulation of the Mus81 complex nuclease activity. Mus81-Mms4 (40 nM) was incubated with circular 3′ flap DNA substrate (6 nM) in the presence or absence of RFC (180 nM), PCNA (70, 140, 270 nM) and ATP (1 mM) as indicated. The reactions were incubated at 37 °C for 30 min and then analyzed on denaturing gel. **b** RFC targets the Mus81 complex in the nicked substrate. Mus81-Mms4 (0.4 nM) was incubated with the 3′ flap substrate (4 nM) in the presence or absence of ΦX174 virion circular ssDNA (0.25 nM), and increasing concentrations of RFC (5, 12.5, 25, and 50 nM), as indicated. Reactions were incubated at 37 °C for 30 min and analyzed. **c** Quantification of data in (b) from three independent experiments. *Raw data provided in Additional file 2

However, to address the effect on nuclease activity of the Mus81 complex when PCNA is loaded on DNA, we performed a nuclease assay in the presence of the circular 3′ flap DNA structure. To monitor the efficient RFC-mediated loading of PCNA on this substrate we used radioactively labeled PCNA in the assay described earlier [41] (Additional file 4: Figure S3C). Under conditions with maximum loading of PCNA we observed a slight but concentration-dependent stimulation of the Mus81 complex nuclease activity (Fig. 5a, lanes 5–7) compared to the reactions with non-loaded PCNA where no effect was detected (Fig. 5a, lanes 8–10). Interestingly, this PCNA-mediated stimulation was observed in a much lower PCNA/Mus81 complex ratio compared to the 3′ flap substrate, suggesting that loaded PCNA has an additive effect to RFC in stimulation of the Mus81 complex. This observation led us to investigate whether RFC can target this enzymatic complex to the DNA substrate. To this end, we performed an experiment with simultaneously added ΦX174 circular ssDNA and linear 3′ flap substrates. While the addition of ΦX174 DNA significantly blocked the cleavage of 3′ flap DNA by the Mus81 complex, the addition of RFC restored the DNA cleavage (Fig. 5b, c; Additional file 2), indicating the ability of RFC to target the Mus81 complex to nick-containing DNA substrate. Moreover, we performed a targeting assay in the presence and absence of ATP. As shown in Additional file 2: Figure S4 and Additional file 5, we observed slightly increased stimulation of the Mus81 complex nuclease activity by the RFC complex in the presence of 1 mM ATP.

### Epistatic relationship of interaction between the Mus81 complex and the RFC complex

**Fig. 6:**
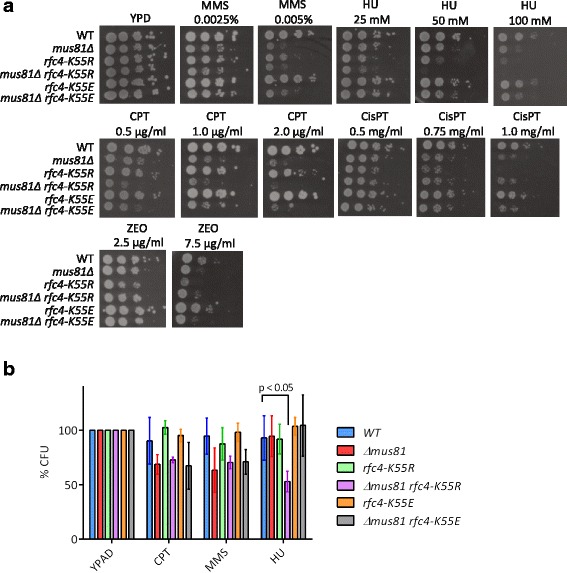
Sensitivity of yeast strains to DNA-damaging agents. **a** Spot test assay. Serial dilutions (10^1^, 10^2^, 10^3^, 10^4^, and 10^5^ cells) of isogenic wild type and single or double mutant yeast strains were spotted on YPD plates containing the indicated concentrations of CPT, HU, MMS, CisPT, or ZEO. **b** Colony formation assay. The diluted cells (biological triplicates for each strain) were spread onto YPAD plates containing selected drugs (CPT (1 μg/mL), MMS (0.0025%), HU (30 mM)) and the colonies were counted after 2–4 days of incubation at 30 °C. *P* values were calculated using two-tailed unpaired homoscedastic *t*-test. *Raw data provided in Additional file 6

To corroborate the cooperation between the Mus81 complex and the RFC complex in vivo, we used a spot assay (Fig. 6a), colony counts (Fig. 6b and Additional file 6), and a cell survival assay (Additional file 7: Figure S5 and Additional file 8) to analyze the epistatic relationship of these two factors with respect to removal of RF damage and DSB repair. As each of the RFC subunits is essential for cell viability, we used a *rfc4-K55R* allele, which has been shown to sensitize cells to MMS and HU. In addition, a *rfc4-K55E* was used as a control as it does not show any significant growth defects in comparison to wild type RFC4 [30]. The *mus81*, *rfc4-K55R*, and *rfc4-K55E*, as well as the respective double mutants, were tested for sensitivity to different DNA-damaging agents, including CPT, HU, MMS, CisPT, and ZEO. As expected, *mus81* mutant cells were sensitive to MMS (Fig. 6 and Additional file 7: Figure S5). *rfc4-K55R* cells, unlike *rfc4-K55E* cells, showed mild sensitivity to MMS. Double *rfc4-K55R mus81* mutant was as sensitive (Fig. 6a), or moderately less sensitive (Fig. 6b and Additional file 7: Figure S5) to MMS as the *mus81* single mutant. On the other hand, double mutant *rfc4-K55E mus81* showed slightly higher resistance to MMS compared to *mus81* single mutant (Fig. 6 and Additional file 7: Figure S5). However, the differences in the individual assays are minimal. In addition, both double mutants showed comparable sensitivity to CPT as the *mus81* single mutant in the colony counting and survival assays (Fig. 6b and Additional file 7: Figure S5), while a marginally increased sensitivity of double mutant *rfc4-K55R mus81* comparing to single *mus81* mutant was detected using the spot test (Fig. 6a). This effect might be explained by the growth defect of the *rfc4-K55R mus81* mutant. Even though the differences between used yeast strains after MMS or CPT treatment are not statistically relevant, these data might support the notion that the Mus81 complex and RFC act together in replication stress caused by the damage generated by MMS and CPT, respectively.

Moreover, *rfc4-K55R* exhibited an increased sensitivity to high concentrations of HU in spot and cell survival assays, respectively (Fig. 6a and Additional file 7: Figure S5). Interestingly, *rfc4-K55R mus81* double mutant cells were as or even more sensitive to HU than either single mutant, which could potentially indicate a separate role for the two factors in dealing with stalled RFs caused by depletion of dNTP pools. However, colony counts, where the *rfc4-K55R mus81* double mutant showed statistically significant difference on HU plates compared to wild-type (*P* < 0.05), revealed a growth defect of this mutant, which probably results in a high sensitivity in the spot assay. Noteworthy, using survival assays in liquid media, we did not observe additional sensitivity of *rfc4-K55R mus81* double mutants. Nevertheless, this could be due to lower sensitization by HU in this experiment.

We also analyzed the effect of CisPT and ZEO using a spot assay, which revealed the highest sensitivity of the *rfc4-K55R* single mutant. Interestingly, this was suppressed by deletion of *MUS81*, which might suggest that the Mus81 complex may generate toxic intermediates in this RFC mutant.

## Discussion

The Mus81 complex plays an important role in the maintenance of genome integrity under conditions of replication stress via processing stalled and/or blocked RFs, and in reinitiating the replication in early mitosis [21–23]. Although the role of the Mus81 complex has recently been studied intensively, it is not yet fully understood how this complex is targeted on sites of actions, nor has it been fully described how components of replication machinery might affect the activity of the Mus81 complex.

In this study, we report on a robust stimulation of the activity of the Mus81 complex by yeast RFC. Moreover, this stimulation is further enhanced by ATPase activity of the RFC complex, by the PCNA loading on the DNA substrate, and by targeting Mus81 to a nick-containing substrate. The last being in agreement with the binding distribution of RFC/PCNA on various substrates [42]. Furthermore, we detected direct physical interaction between the Mus81 complex and PCNA, and showed that the interaction is mediated by the PIP box. In addition, the experiment with mutant forms of PCNA revealed that the IDCL domain on PCNA is responsible for Mus81 binding. This domain serves as a pocket for the hydrophobic residues of PIP box motif, further supporting the role of the PIP box motif on Mus81p in PCNA binding. Further biochemical analysis nevertheless suggested that the interaction with PCNA might play a minor role in recruiting and/or stabilizing the Mus81 complex onto the DNA substrate, in particular when loaded on the DNA substrate. Indeed, PCNA is known to bind a variety of proteins to ensure their presence at the active centers of DNA replication and repair [43]. It targets Exo1 to the ssDNA gaps left behind the RF [44]. Similarly, Rad27 was shown to interact with PCNA [45]. Moreover, mutations within the PIP box of FEN1, the human orthologue of Rad27, abolished FEN1 recruitment to RF, which is associated with severe defects in replication progression and may lead to carcinogenesis [46]. Since FEN1 is stimulated by the human RFC complex [40], we compared the effect of RFC on both the Mus81 complex and Rad27. We observed that the activity of Rad27 is robustly stimulated by the RFC complex, confirming the preservation and importance of this stimulation between species. Interestingly, the enzymatic activity of the Mus81 complex was stimulated by the RFC complex to an even higher extent compared to Rad27 stimulation. Moreover, human MUS81-EME1 was also stimulated by yeast RFC, thus confirming that the stimulation is not species-specific.

The fate of the RFC complex after PCNA loading onto a primer-template junction is, at present, unclear. Several studies suggest that RFC dissociates from DNA once PCNA is loaded onto the primer-template junction [29]. However, contradictory evidence suggests that the RFC complex travels along the DNA as part of replication machinery [40]. The second scenario is supported by our findings that the RFC complex effectively stimulates the Mus81 complex. As it moves along the DNA substrate during replication, it would be available to stimulate the Mus81 complex nuclease activity. Consistent with this proposed explanation, we demonstrate the interaction between Mus81 and the RFC complex in an in vivo pull-down experiment. Further experiments with a truncated form of Mus81 (1–319) revealed that the interaction site is situated at the N-terminus of Mus81. Although it cannot be excluded that other factor(s) may mediate this interaction, we assume a direct, albeit transient, interaction between the Mus81 complex and RFC. PCNA could play a role as a mediator of this interaction as the N-terminus of Mus81 also contains a PIP box motif (Fig. 1a).

In the cells, three additional complexes (termed RFC-like complexes in which the largest subunit is exchanged for either Ctf18, Rad24, or Elg1) exist. We wondered whether these complexes could also play a role in activation of the Mus81 complex. Here, we also show that the robust stimulation of the Mus81 complex is not specific only to the RFC complex, since the Elg1-RFC-like complex was also able to stimulate nuclease activity of the Mus81 complex. Although we cannot exclude the possibility that both Rfc1 and Elg1 contain the Mus81 interaction motif, since all subunits of the RFC complex are highly homologous in certain amino acid sequences called RFC boxes (box II–VII), we think that the stimulatory motif might be conserved within all subunits of this complex. Fittingly, a short motif localized in box VII of human RFC subunits has been found to be responsible for the stimulation of human FEN1 [40]. It is therefore possible that alternative clamp loaders/unloaders also play a role in targeting and/or stabilizing the Mus81 complex onto the DNA and stimulating its activity. Interestingly, Bellaoui et al. [47] demonstrated synthetic lethality between Mus81 and Elg1, indicating that Elg1 may represent alternative targeting complex for Mus81. This notion may be also supported by synthetic lethality observed between Elg1 and RFC subunits [48].

The relevance of the interaction between the RFC complex and the Mus81 complex was also verified in vivo using various assays. Single and double mutant strains were tested for sensitivity to different DNA-damaging drugs. We observed a comparable growth defect of the double *rfc4-K55R mus81* mutant and a single mutant carrying the deletion of *MUS81* when the cells were exposed to CPT and to some extent also to MMS. These data might suggest that RFC and the Mus81 complex are involved in the same pathway as well as their possible cooperation in resolution of recombination intermediates and DNA lesions caused by these drugs. Interestingly, the sensitivity of *rfc4-K55R* mutant to CisPT and ZEO was suppressed by deletion of *MUS81*, which might indicate that the Mus81 complex generates toxic intermediates in this RFC mutant. On the other hand, both mutations resulted in a synergistic phenotype after the cells were treated with HU, indicating separate roles of Mus81 and the RFC complex under these conditions. This result is supported by a study showing that the Mus81 complex is not necessary for DNA replication resumption after HU treatment [49]; however, it could act independently of RFC in the later steps of repair.

**Fig. 7:**
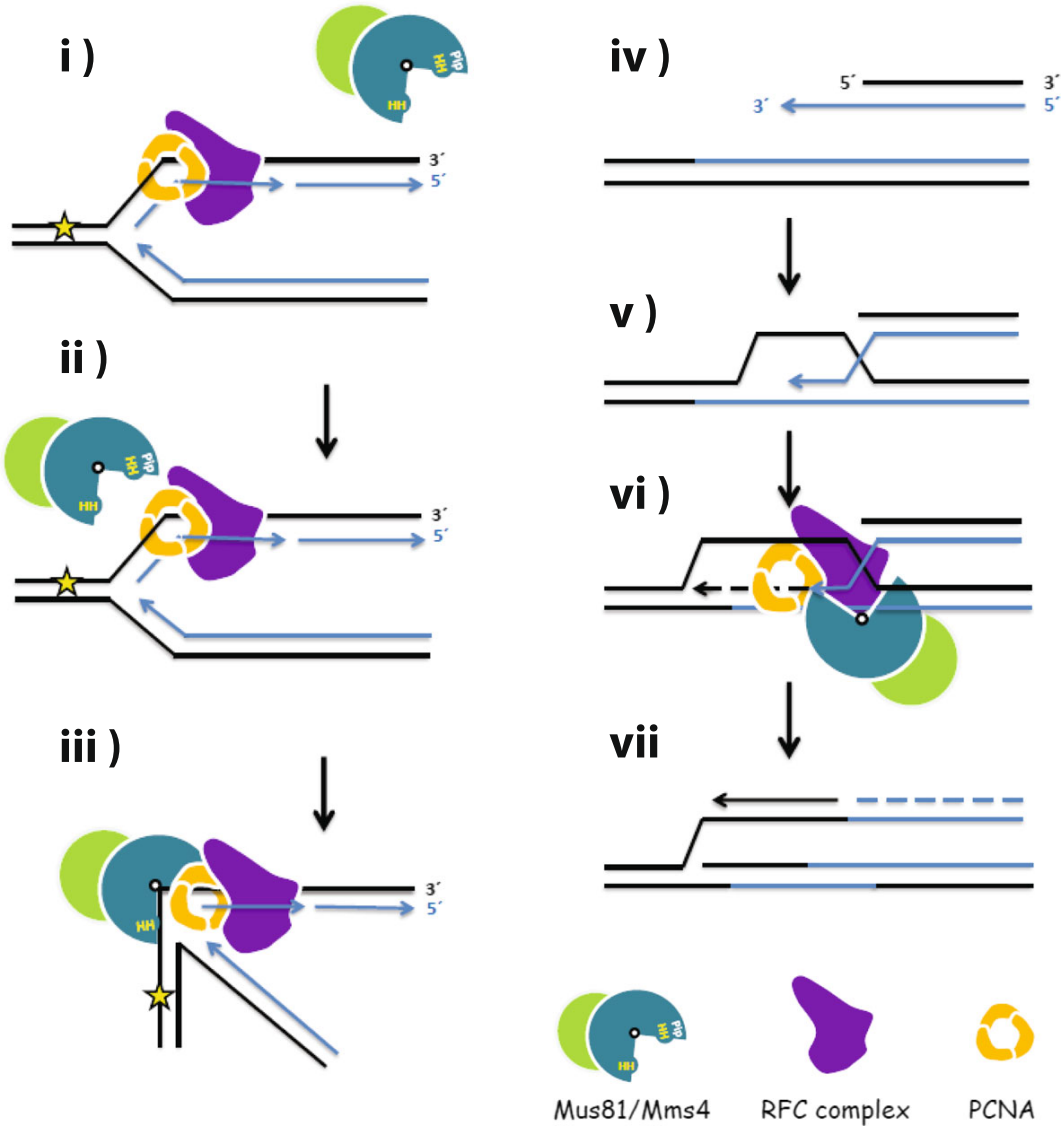
Proposed role of Mus81-Mms4 interaction with RFC and PCNA. Model demonstrating importance of the interaction of the Mus81 complex with PCNA and RFC. **i–ii** Possible recruitment of the enzymatic complex to the damage site by interaction with PCNA. **iii** Stabilization of the Mus81 complex in the active conformation together with the RFC complex. **iv–v** Further steps of repair after DSB formation include homologous recombination-mediated single-end invasion followed by cleavage of sister chromatid junction, (**vi**) restoring replication or BIR

Based on the known facts and the data presented in this work, we propose a model of the role of the interaction between the Mus81 and RFC complexes in restart of RFs during recovery from replication stress (Fig. 7). DNA-damaging agents inducing replication stress frequently cause stalling and/or collapse of RFs. In order to maintain genome stability, the stalled forks need to be dealt with efficiently. Here, we assume that the Mus81 complex may be targeted to the damage site through the N-terminus containing the PCNA interaction motif [50]. Recent work has shown that the Mus81 complex undergoes conformational changes and bends its DNA substrate for cleavage [51, 52]. A scenario reminiscent of FEN1 and EXO1 nucleases [53]. We propose that the interaction with RFC might play an important role in stabilizing the Mus81 complex in its active conformation (Fig. 7, iii). After the Mus81 complex, or other means, generate broken RFs, the BIR repair pathway where homology is restricted to only one end via homology-mediated invasion is required (Fig. 7, iv–v). However, the BIR is characteristic for high levels of template switches and point mutations [25, 54]. The mutation rates are significantly increased near the broken site but drop to spontaneous levels at a distance from a DSB [24], indicating a shift in the fidelity of the synthesis during repair. In this context, the Mus81 complex has been implicated in processing recombination intermediates during BIR and to suppress template switches during BIR by cleavage of the D-loop structure, thus limiting error-prone synthesis and promoting POLD3-dependent DNA synthesis [23, 26, 55, 56]. Moreover, all major replicative polymerases whose proper activity is dependent on RFC/PCNA are involved in this pathway, thus ensuring their presence and accessibility for the Mus81 complex during BIR synthesis [57].

## Conclusions

In summary, we provide evidence that the Mus81 complex, as an important factor involved in resolution of stalled and/or blocked RFs, is targeted and stabilized in the place of its action by the PCNA/RFC complex. We believe our data help shed light onto the complex interplay of different repair mechanisms that take place during DNA replication. Additionally, it has been shown that replicative stress plays a key role in tumorigenesis [58]; our results point to new options for control of the repair mechanisms and the subsequent treatment of disorders associated with genome instability.

## Methods

### Yeast strains and plasmids

The *S. cerevisiae* strains and plasmids used in this study are listed in Additional file 9: Table S1. All strains used for drug sensitivity assays are derivatives of W1588-4C (*MATa ade2-1 can1-100 ura3-1 his3-11, 15 leu2-3,112 trp1-1*) [59], a RAD5 derivative of W303-1A [60]. Standard genetic techniques were used for manipulating yeast strains [61]. The *Δmus81* strain was created by replacing the gene coding sequence with the *HIS5*-disruption cassette in the wild-type strain. The correct integration was verified by PCR reaction. The yeast strains *rfc4-K55R* and *rfc4-K55E* were generated by transformation of plasmids pBL625 or pBL633-E into the strain PY94 (generous gifts from Dr. Peter Burgers). The mutants were selected on plates with 5-fluoroorotic acid. MATα *rfc4* mutant strains were prepared by crossing wild-type and MATa *rfc4-K55R* or *rfc4-K55E*. Crossing haploid *Δmus81* and the particular *rfc4* single mutant strains generated corresponding double mutant strains. The heterozygous diploids were sporulated and the tetrads were dissected. Double mutant haploid strains were subsequently verified using PCR and selected for specific markers.

The plasmid for expression of Mus81 (1–319) E44Q mutant was prepared by site-directed mutagenesis using specific primers (Additional file 10: Table S2).

### Expression and purification of Mus81-Mms4 (MUS81-EME1)

The yeast Mus81-Mms4 and human MUS81-EME1 expression plasmids were a generous gift from Dr. Matthew Whitby (University of Oxford) and Dr. Steve West (The Francis Crick Institute), respectively. Expression and purification of these complexes has been described elsewhere [18]. Briefly, lysate was prepared from 40 g of *E. coli* cell paste using sonication in 200 mL of lysis buffer C (50 mM Tris-HCl (pH 7.5), 10% sucrose, 10 mM EDTA, 1 mM β-mercaptoethanol, 0.01% Nonidet P-40, and protease inhibitors (aprotinin, chymostatin, leupeptin, pepstatin A, benzamidine, each at 5 μg/mL)) containing 150 mM KCl. Cleared lysate was applied sequentially onto a 20-mL Q-Sepharose and a 20-mL SP-Sepharose column (GE Healthcare). Proteins were eluted from the SP-Sepharose column with a 200-mL gradient from 150 to 1000 mM KCl in buffer K (20 mM K_2_HPO_4_ (pH 7.5), 10% sucrose, 10 mM EDTA, 1 mM β-mercaptoethanol, and 0.01% Nonidet P-40). The peak fractions were pooled and mixed with 1 mL of His-Select nickel affinity gel (Sigma-Aldrich) for 1 h at 4 °C. The beads were washed with 10 mL of buffer K containing 150 mM KCl and 10 mM imidazole. The bound proteins were eluted using 50, 150, 300, and 500 mM imidazole in buffer K containing 150 mM KCl. The 150, 300, and 500 mM imidazole fractions were pooled and loaded onto a 1-mL Heparin column (GE Healthcare). Bound proteins were eluted with a 10-mL gradient from 150 to 1000 mM KCl in buffer K. Peak fractions were pooled, loaded onto a 1-mL Mono S column (GE Healthcare), and eluted with a 10-mL gradient from 150 to 1000 mM KCl in buffer K. Pooled fractions were concentrated, flash-frozen in 5 μL aliquots, and stored at −80 °C.

### Expression and purification of Mus81 (1–319)

The *E. coli* expression construct of Mus81 (1–319) was a kind gift from Dr. Steven Brill (Rutgers University). The expression and purification of Mus81 (1–319) was based on a procedure previously described [24]. Extract from 10 g of cell paste was prepared by sonication in 50 mL of lysis buffer C containing 150 mM KCl and clarified by centrifugation. The supernatant was passed through a 7-mL Q-Sepharose column and the flow-through was directly applied onto a 7-mL SP-Sepharose. Proteins were eluted with a 70-mL gradient from 150 to 1000 mM KCl in buffer K. Mus81 (1–319) peak fractions were pooled and mixed with His-Select nickel affinity gel (Qiagen) for 1 h at 4 °C. The bound proteins were eluted using 500 μL of each 150, 300, and 500 mM imidazole in buffer K containing 150 mM KCl. Peak fractions were again pooled and loaded onto a 1-mL Mono S column. Mus81 (1–319) was eluted using 10-mL gradient from 150 to 1000 mM KCl in buffer K. The fractions containing nearly homogeneous Mus81 (1–319) were concentrated, flash-frozen, and stored in small portions at −80 °C.

### Expression and purification of Mus81 (1–319) E44Q

The plasmid expressing Mus81 (1–319) mutant was transformed into *E. coli* BL21(DE3) strain and protein expression was induced with 0.1 mM IPTG followed by incubation at 16 °C overnight. The cell pellet (8 g) was resuspended in 30 mL of lysis buffer C containing 150 mM KCl. After sonication, the cell lysate was clarified by ultracentrifugation. The supernatant was incubated with 1 mL of His-Select nickel affinity gel (Sigma-Aldrich) for 1 h at 4 °C. The beads were washed with 10 mL of buffer K containing 150 mM KCl. The bound proteins were eluted using 50, 150, 300, 500, and 1000 mM imidazole in buffer K containing 50 mM KCl. Peak fractions were pooled and applied onto a 1.5-mL Source S column. The protein was eluted with a 12-mL gradient from 250 to 1000 mM KCl in buffer K. Fractions containing Mus81 protein were concentrated, flash-frozen, and stored in small portions at −80 °C.

### Expression and purification of PCNA, PCNA-79, and PCNA-90

The *E. coli* strain BL21(DE3), transformed with expression plasmid containing untagged PCNA, PCNA-79, or PCNA-90 (a kind gift from Dr. Peter Burgers, Washington University) was induced with 0.5 mM IPTG for 3 h at 37 °C. Cells were harvested by centrifugation and stored at −80 °C. Wild-type and mutant forms of PCNA were purified as described elsewhere [62]. Briefly, 5 g of *E. coli* cell paste were sonicated in 30 mL lysis buffer C containing 750 mM KCl. The lysate was clarified by ultracentrifugation (100,000 × *g* for 1 h at 4 °C) and subjected to ammonium sulfate precipitation at 0.21 g/mL. After stirring for 1 h and centrifugation at 15,000 × *g* for 20 min at 4 °C, another 0.32 g/mL of ammonium sulfate was added to the supernatant. The resulting precipitate was dissolved in 50 mL of buffer K and the mixture was applied onto a 7-mL Q-Sepharose column. Proteins were eluted with a 70-mL linear gradient from 50 to 1000 mM KCl in buffer K. The fractions containing PCNA were pooled and loaded onto a 1-mL hydroxyapatite column (BioRad) and proteins were eluted with a 10-mL gradient from 50 to 1000 mM K_2_HPO_4_ in buffer K. Peak fractions were pooled and loaded onto a 1 mL Mono Q column (GE Healthcare) and proteins were eluted with a 10-mL gradient from 50 to 1000 mM KCl in buffer K. Fractions with purified PCNA were pooled and concentrated in a Vivaspin micro concentrator. Small aliquots (5 μL) were stored at −80 °C.

### Expression and purification of RFC complexes

The expression constructs for RFC1 and ELG1 complexes were a kind gift from Dr. Jeff Finkelstein (Rockefeller University) and Dr. Peter Burgers (Washington University), respectively, and they were expressed accordingly [62]. For RFC complex purification, 10 g of cell pellet was lysed in a CryoMill (Retsch) and the resulting powder was resuspended in 50 mL lysis buffer C containing 100 mM KCl, followed by sonication. The lysate was clarified by ultracentrifugation (100,000 × *g* for 1 h at 4 °C) and applied to a 7-mL SP-Sepharose column. The bound proteins were eluted with a 70-mL linear gradient from 100 to 1000 mM KCl in buffer K. The peak fractions were pooled and loaded onto a 1-mL Mono Q column (GE Healthcare) and the proteins were eluted with a 10-mL gradient from 100 to 900 mM KCl in buffer K. The nearly homogeneous RFC complex was concentrated and stored in small portions at −80 °C.

### Elg1-RLC purification

Elg1-RLC was purified according to the protocol [63], with minor modifications. The *S. cerevisiae* BJ5464 strain was transformed with plasmids pBL448 (encoding GST-ELG1 under control of GAL1 promoter) and pBL422 (encoding RFC2, RFC3, RFC4, and RFC5 under control of GAL1 promoter). These materials were generously provided by Dr. Peter Burgers. To overexpress Elg1-RLC, an overnight yeast culture grown in synthetic medium lacking tryptophan and uracil was diluted eight-fold with fresh synthetic medium lacking tryptophan and uracil but containing galactose (2% w/v), glycerol (3%), and lactic acid (3%). The culture was incubated for 22–24 h at 30 °C with shaking. Cells were harvested by centrifugation and stored at −80 °C.

Elg1-RLC was purified by opening up 70–100 g of yeast paste by cryogenic milling. The resulting powder was dissolved in 200 mL of lysis buffer C1 (50 mM Tris-HCl (pH 7.5), 10% sucrose (w/v), 10 mM EDTA, 3 mM dithiothreitol, 0.01% Nonidet P-40, 150 mM NaCl, and the protease inhibitors) and the volume of the crude lysate was measured. Solid ammonium sulfate was then added to the lysate to a final concentration of 300 mM and the mixture was stirred for 25 min at 4 °C. Afterwards, 45 μL of 10% Polymin P per 1 mL of the lysate was added, and the mixture was gently stirred at 4 °C for 10 min. The crude lysate was clarified by centrifugation (100,000 × *g* for 90 min). Next, the Elg1-RLC complex was precipitated from the cleared lysate using ammonium sulfate (0.35 g/mL), and the precipitate was held overnight at −80 °C. The next day, the precipitate was dissolved in buffer T (25 mM Tris-HCl, 10% (v/v) glycerol, 5 mM EDTA, pH 7.5). The conductivity of the sample was then checked to be equal to that of buffer T containing 150 mM NaCl and mixed with 2 mL of glutathione sepharose (GE Healthcare) equilibrated in the same buffer. After 2–3 h at 4 °C, the beads were washed with 150 mL of buffer T containing 150 mM NaCl. GST-Elg1-RLC was eluted with buffer T containing 150 mM NaCl, supplemented with 20 mM glutathione (reduced form) and 0.05% ampholytes. Fractions containing the complex were pooled and incubated for 3 h at 4 °C with 5–15 μg of PreScission protease (GE Healthcare). The Elg1-RLC complex was then loaded onto a 1-mL Mono S column equilibrated with buffer T containing 100 mM NaCl and 0.05% ampholytes. After a 10-mL wash with buffer T containing 100 mM NaCl, 0.05% ampholytes, 5 mM MgCl_2_, and 100 μM ATP, the complex was eluted with a 20-mL linear gradient from 100 to 500 mM NaCl. Fractions containing Elg1-RLC were pooled, concentrated in a Vivaspin concentrator, then stored in 2 μL aliquots at −80 °C.

### Expression and purification of other proteins

Rad27 nuclease was purified according to a procedure described previously [18]. PCNApka was purified as described previously [41].

### DNA substrates

The DNA substrates used in this study were prepared using the synthetic oligonucleotides listed in Additional file 10: Table S2. All oligonucleotides were purchased from VBC Biotech. Oligo1 is fluorescently labeled at the 5′ end. The substrates were prepared as described elsewhere [64]. Briefly, equimolar amounts of individual oligonucleotides were annealed in hybridization buffer H (50 mM Tris–HCl (pH 7.5), 100 mM NaCl, and 10 mM MgCl_2_). The mixture was heated to 90 °C for 3 min and then cooled down slowly to room temperature. The annealed DNA substrates were purified by fractionation on a 1-mL Mono Q column (GE Healthcare) with a 20-mL gradient of 50 to 1000 mM NaCl in 10 mM Tris–HCl (pH 7.5). Peak fractions were filtered, dialyzed into 50 mM Tris–HCl (pH 7.5), containing 5 mM MgCl_2_, then concentrated in a Vivaspin concentrator with a 5 kDa cutoff. The concentration of the DNA substrates was determined by absorbance measurement at 260 nm.

### Nuclease assay

Nuclease assays were performed as previously described [18]. Briefly, reactions containing purified Mus81 complex were performed with 5 nM DNA substrate in buffer N (20 mM Tris–HCl (pH 7.5), 0.2 mM DTT, 10 mM MgCl_2_, and 5% glycerol). For the human MUS81-EME1 complex, the buffer used contained 50 mM Tris–HCl (pH 7.5), 5 mM MgCl_2_, 1 mM DTT, and 100 μg/mL BSA. After incubation for 20 min at 37 °C, the reaction was stopped by addition of 0.1% SDS and 500 μg/mL of proteinase K and additional incubation for 5 min at 37 °C. The reactions were mixed with 1/10 volume of loading buffer (60% glycerol, 10 mM Tris-HCl (pH 7.4), 60 mM EDTA, and 0.1% Orange G). The fluorescent DNA was visualized by FLA-9000 (Fuji) and quantified using Multi-Gauge V3.2 (Fuji) software. Rad27 reactions were performed as described above, except that the buffer contained 50 mM Tris–HCl (pH 7.5), 1 mM DTT, and 5 mM MgCl_2_.

In the Mus81 complex targeting assay, 4 nM 3′ flap substrate and 0.25 nM ΦX174 virion circular ssDNA were mixed in N buffer together with 0.4 nM Mus81-Mms4 and increasing concentrations of RFC (5, 12.5, 25, 50 nM). The mixture was incubated at 37 °C for 30 min and analyzed as described above.

### Nuclease assay (with circular 3′ flap)

RFC (180 nM) was pre-incubated with circular 3′ flap DNA substrate (6 nM) and increasing concentrations of PCNApka (70, 140, 270 nM) in the presence or absence of ATP (1 mM) in buffer REP (20 mM Tris–HCl pH 7.5, 1 mM DTT, 12 mM MgCl_2_, 100 mM KCl) at 30 °C for 10 min. Then, Mus81-Mms4 (40 nM) was added to the reactions followed by incubation at 37 °C for 30 min. The reaction was stopped by addition of 0.05% SDS and 0.5 μg/μL proteinase K and additional incubation at 37 °C for 10 min. The reactions were mixed with a loading dye (45% formamide, 0.025% Orange G), boiled for 3 min at 95 °C, and analyzed on 12% acrylamide denaturing gel. The fluorescent DNA was visualized and analyzed as described above.

### PCNA loading assay

Phosphorylated PCNApka was prepared as described previously [41]. PCNA loading was analyzed on a circular 3′ flap substrate (6 nM, created by annealing of ΦX174 virion circular ssDNA with pR3276 and fluorescently labeled pR3277) in buffer REP. RFC (180 nM) was incubated with ^32^P-PCNApka (140/270 nM) in the presence or absence of ATP (1 mM) at 30 °C for 10 min. Then, 0.02% glutaraldehyde was added to the reactions followed by additional incubation at 37 °C for 10 min. The reaction was stopped by addition of loading dye (60% glycerol, 10 mM Tris-HCl pH 7.4, 60 mM EDTA, and 0.025% orange G) and resolved on 0.9% agarose gel in 0.5× TBE buffer. After electrophoresis, the gel was dried on Grade 3 CHR paper (Whatman), exposed to a phosphoimager screen, and scanned using Fuji FLA 9000 imager.

### Affinity pull-down

The Mus81 complex was captured using Ni-NTA beads (Bio-Rad) specific for His_6_-tag on Mms4 protein. The purified Mus81 complex (10 μg) was incubated with yeast extract (150 μL) containing FLAG-tagged Rfc2 and Rfc4, respectively. The reactions were mixed with 20 μL of Ni-NTA beads for 30 min at 4 °C. The supernatant was then removed and the beads were washed twice with 150 μL of IP150 buffer (50 mM Tris–HCl (pH 7.5), 150 mM KCl, 0.5% Triton X-100, and protease inhibitors). The bound proteins were eluted with 30 μL of 5% SDS. Supernatant and bead fractions (10 μL of each) were analyzed on SDS-PAGE followed by immunoblotting using anti-FLAG (1:6000 dilution, Sigma-Aldrich Cat# F3165 RRID:AB_259529) and anti-His antibodies (1:8000 dilution, Sigma-Aldrich Cat# H1029 RRID:AB_260015), respectively, and secondary anti-mouse antibodies (1:40000 dilution, Sigma-Aldrich Cat# A0168 RRID:AB_257867).

PCNA or BSA was conjugated with Affi-beads (Bio-Rad) according to the manufacturer’s protocol. Purified Mus81 complex was mixed with 10 μL of beads in 30 μL of Tris buffer containing indicated concentration of KCl and the reaction was incubated for 30 min at 4 °C. The beads were washed twice with reaction buffer and bound proteins were eluted with 30 μL of 5% SDS.

### Microscale thermophoresis

Binding affinity quantifications via microscale thermophoresis were performed using the Monolith NT.115 instrument (NanoTemper Technologies). The purified PCNA protein was labeled using the L001 Monolith NT.115 protein labeling kit RED-NHS (Amine Reactive) dye. Affinity measurements were performed using MST buffer (T buffer containing 0.05% Tween-20 and 150 mM KCl). Samples were soaked into NT.115 standard treated capillaries (MO-K002, NanoTemper Technologies). Measurements were performed at 25 °C, 70% LED, 40% IR laser power, constant concentration of labeled PCNA (22 nM) and increasing concentration of Mus81 fragment (1.3–5500 nM). Data were analyzed by the MO.Affinity Analysis software (NanoTemper Technologies).

### Sensitivity assays

For spot analysis, yeast strains were inoculated in YPD liquid medium and grown overnight at 30 °C. The following day, the overnight culture was diluted to OD_600_ = 0.2 and grown for an additional 3 h. The concentration of each culture was adjusted to OD_600_ = 0.2 and serial dilutions were performed in order to reach a corresponding cell density (1 × 10^5^, 1 × 10^4^, 1 × 10^3^, 1 × 10^2^, 1 × 10^1^ cell/mL). From each of these dilutions, 3 μL were spotted onto YPD plates containing HU, CPT, MMS, CisPT, or ZEO, respectively, at the indicated concentrations. Images were taken after 2 days of incubation at 30 °C.

For colony formation assay, the diluted cells were spread onto YPAD plates containing selected drugs and the colonies were counted after 2–4 days of incubation at 30 °C. Three repeats were performed and the means and standard deviations were calculated. The statistical analysis was performed using a two-tailed unpaired homoscedastic *t*-test.

### Cell survival assay

For analysis in liquid medium, the yeast strains were inoculated in 3 mL YPAD at 30 °C overnight. The next day, cultures were diluted to OD_600_ 0.1 and grown for an additional 2.5 h at 30 °C. Then, the selected drugs (MMS, CPT and HU) were added to each culture and growth was monitored by OD_600_ measurement at specific time points. Three biological replicates were performed for each strain. For quantification, the OD_600_ of each culture was normalized to the OD_600_ value of the untreated culture at the same time point.

## Additional files

Additional file 1: Figure S1. Interaction of Mus81-Mms4 with PCNA and PCNA-dependent stimulation of Mus81-Mms4 activity. (A) Purified proteins used in this study. (B) Purified recombinant Mus81-Mms4 (5 μg) was mixed with PCNA covalently bound to Affi-beads in Tris buffer containing 150 mM KCl in the presence (lanes 3–6) or absence (lanes 1 and 2) of short peptides: pFF representing PIP box motif (QxxLxxFF) or pAA representing PIP box with mutation (QxxLxxAA). After 30 min incubation at 4 °C, the supernatant was removed and the beads were washed twice with Tris buffer containing 150 mM KCl. The unbound (U) and bound (B) fractions were then analyzed on 12% SDS gel. (C–E) Time course enhancement of the Mus81 complex nuclease activity by PCNA on various DNA substrates. Mus81-Mms4 (0.2 nM) was incubated with the indicated DNA substrates (4 nM) in the presence or absence of PCNA (0.5 μM). Reactions were incubated at 37 °C for 60 min. Aliquots of the reactions were taken at the indicated times and analyzed. (PDF 2791 kb)

Additional file 2: The individual data values for all nuclease assays. (XLSX 21 kb)

Additional file 3: Figure S2. Stimulation of Mus81-Mms4 nuclease activity by RFC complex. (A) RFC stimulation of human MUS81-EME1 complex. Reaction mixtures containing DNA substrate (5 nM) and the Mus81-Mms4 (0.25 nM, lanes 1–5) or MUS81-EME1 (0.25 nM, lanes 6–10) were incubated with increasing amounts of RFC complex (0.25, 0.5, 1.25, and 2.5 nM) at 37 °C for 20 min and then analyzed. (B–G) Time course enhancement of the Mus81 complex nuclease activity by RFC on various DNA substrates. Mus81-Mms4 (0.2 nM) was incubated with indicated DNA substrates (4 nM) in the presence or absence of RFC (2 nM). Reactions were incubated at 37 °C for 60 min. Aliquots of the reactions were taken at the indicated times and analyzed. Quantification of data from three independent experiments was performed for each DNA substrate. *Raw data provided in Additional file 2. (PDF 2850 kb)

Additional file 4: Figure S3. Effect of RFC and PCNA on nuclease activity of the Mus81 complex*.* (A) Effect of PCNA (0.25, 0.5, 1.25, and 2.5 nM or 0.05, 0.1, 0.25, and 0.5 μM) in the presence of RFC (0.25, 0.5, 1.25, and 2.5 nM) on the nuclease activity of the Mus81 complex. Standard endonuclease assay (37 °C, 20 min) was performed with the indicated amounts of enzyme. (B) Quantification of products formed in panel A. (C) PCNA is efficiently loaded on circular 3′ flap substrate. Loading reaction of ^32^P-PCNA (140, 270 nM) on circular 3′ flap DNA substrate (6 nM) was performed in the presence or absence of RFC (180 nM) and ATP (1 mM) as indicated. *Raw data provided in Additional file 2. (PDF 1762 kb)

Additional file 5: Figure S4. Effect of ATP on substrate targeting of the Mus81 complex by RFC. (A) Mus81-Mms4 (0.4 nM) was incubated with 3′ flap substrate (4 nM) in the presence or absence of ΦX174 virion circular ssDNA (0.25 nM), ATP (1 mM), and increasing concentrations of RFC (5, 12.5, 25, 50 nM). Reactions were incubated at 37 °C for 30 min and analyzed. (B) Quantification of the data in A from three independent experiments. *Raw data provided in Additional file 2. (PDF 1432 kb)

Additional file 6: The individual data values for colony formation assay. (XLSX 22 kb)

Additional file 7: Figure S5. Analysis of cell growth in liquid medium. Yeast cells (biological triplicates for each strain) were treated by various DNA-damaging agents (CPT (5 μg/mL), MMS (0.01%), HU (50 mM)) and the cell growth was analyzed by OD_600_ measurement at the indicated times. *Raw data provided in Additional file 8. (PDF 932 kb)

Additional file 8: The individual data values for cell survival assay. (XLSX 36 kb)

Additional file 9: Table S1. (A) Strains and (B) plasmids used in this study. (DOC 36 kb)

Additional file 10: Table S2. Oligonucleotides used in this study together with the structures of DNA substrates. Fluorescence is marked by an asterisk. (DOC 49 kb)
