# Supplementary figures and images for: Role of PCNA and RFC in promoting Mus81-complex activity
Source: BMC Biol. 2017 Oct 2;15:90. doi: 10.1186/s12915-017-0429-8 (PMC5625722; doi:10.1186/s12915-017-0429-8)

# Supplementary Figure 1

**A**

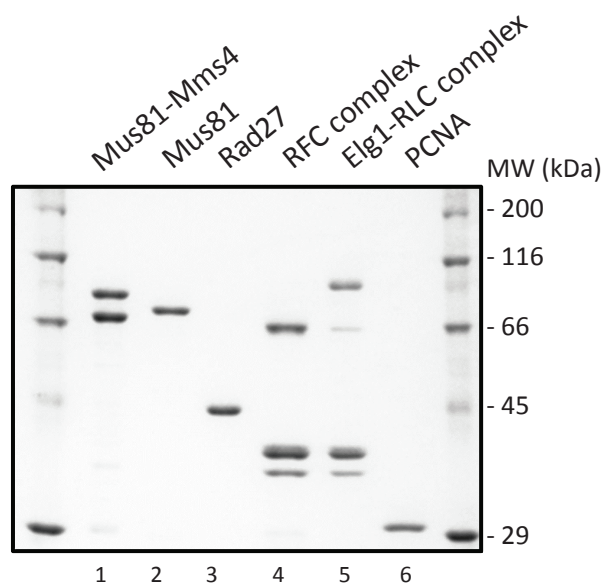

**B**

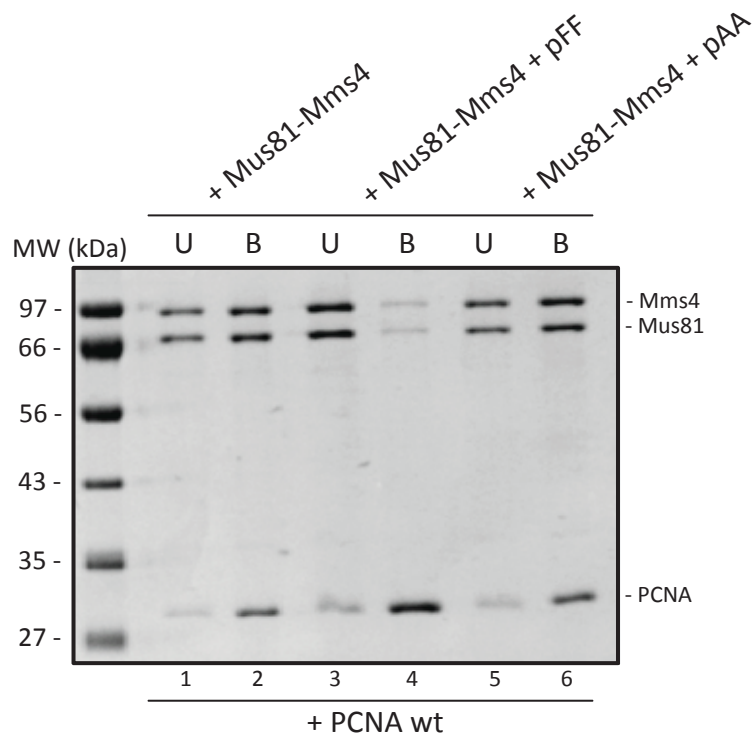

**C**

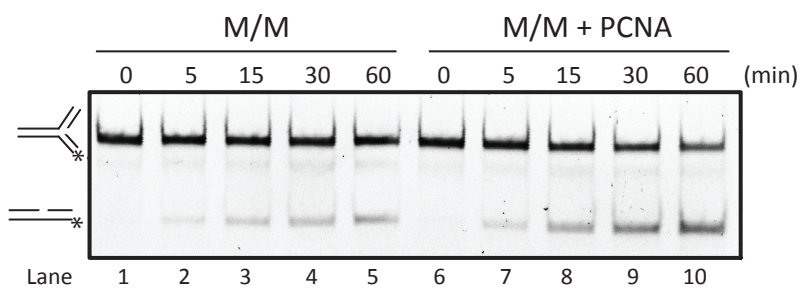

**D**

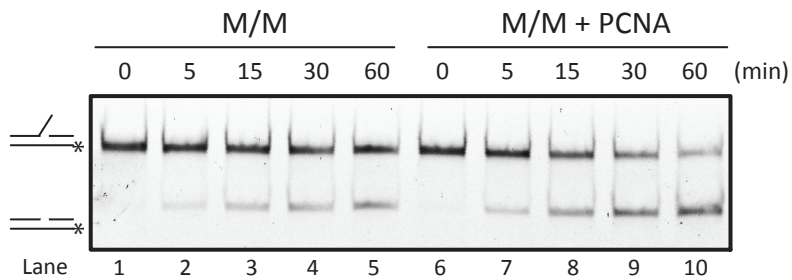

**E**

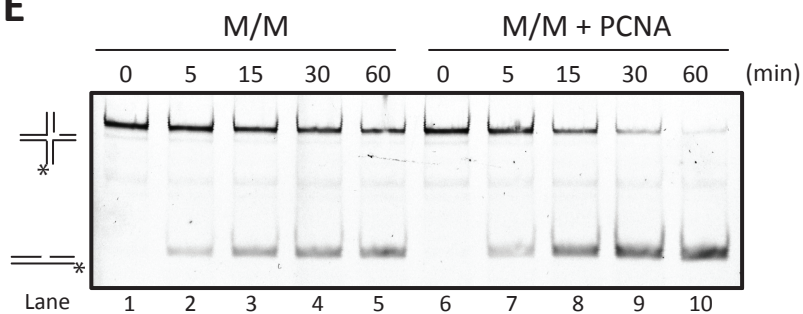

Supplement: Supplementary file 1 — Interaction of Mus81-Mms4 with PCNA and PCNA-dependent stimulation of Mus81-Mms4 activity. (A) Purified proteins used in this study. (B) Purified recombinant Mus81-Mms4 (5 μg) was mixed with PCNA covalently bound to Affi-beads in Tris buffer containing 150 mM KCl in the presence (lanes 3–6) or absence (lanes 1 and 2) of short peptides: pFF representing PIP box motif (QxxLxxFF) or pAA representing PIP box with mutation (QxxLxxAA). After 30 min incubation at 4 °C, the supernatant was removed and the beads were washed twice with Tris buffer containing 150 mM KCl. The unbound (U) and bound (B) fractions were then analyzed on 12% SDS gel. (C–E) Time course enhancement of the Mus81 complex nuclease activity by PCNA on various DNA substrates. Mus81-Mms4 (0.2 nM) was incubated with the indicated DNA substrates (4 nM) in the presence or absence of PCNA (0.5 μM). Reactions were incubated at 37 °C for 60 min. Aliquots of the reactions were taken at the indicated times and analyzed. (PDF 2791 kb) [file 12915_2017_429_MOESM1_ESM.pdf]

Supplementary Figure 2

A

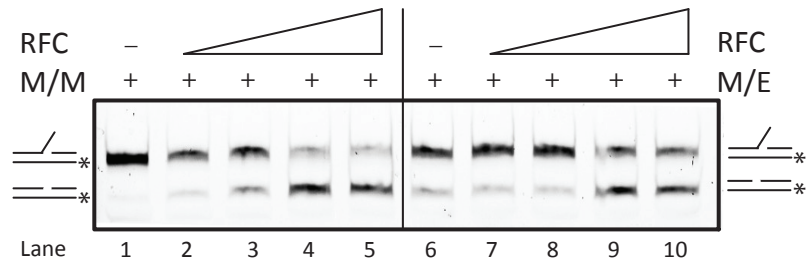

B

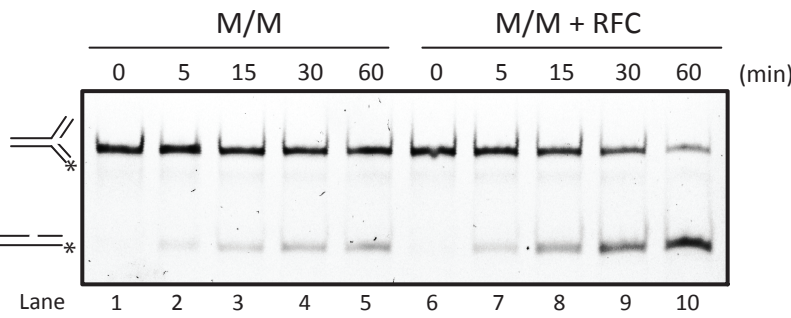

C

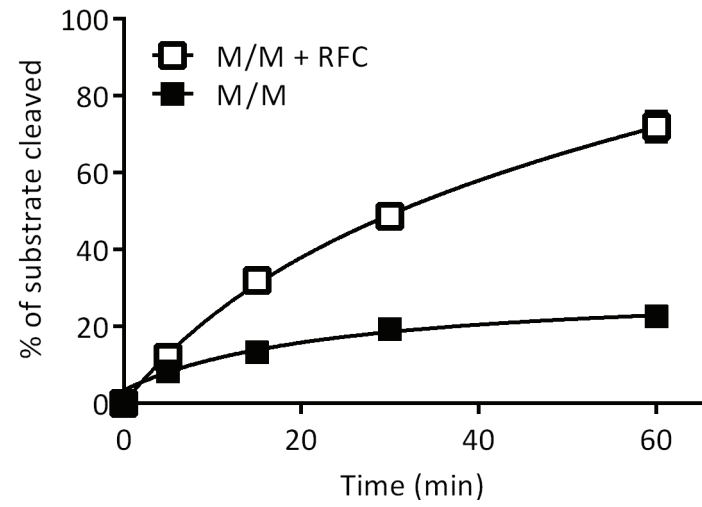

D

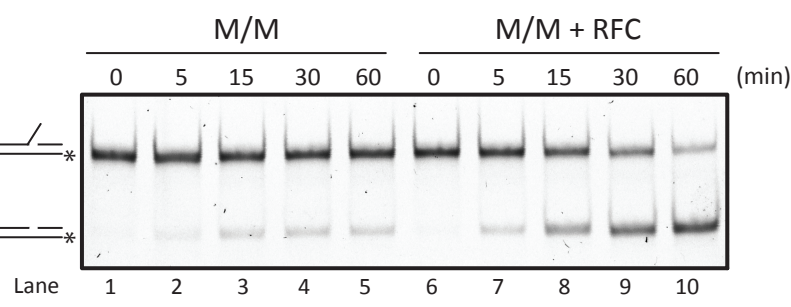

E

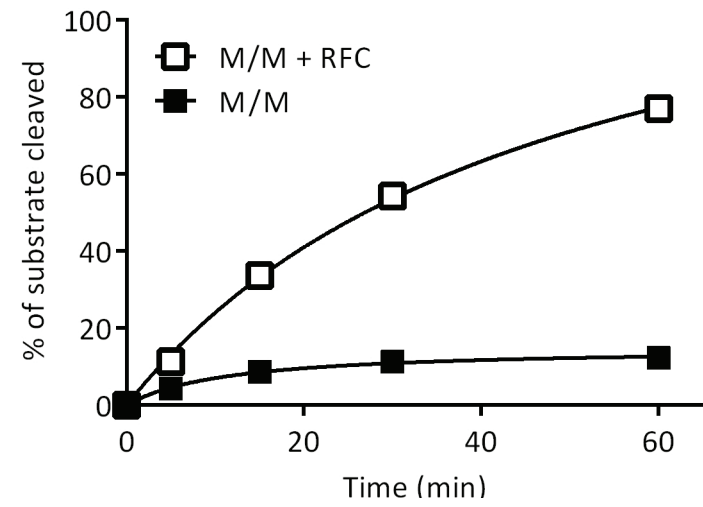

F

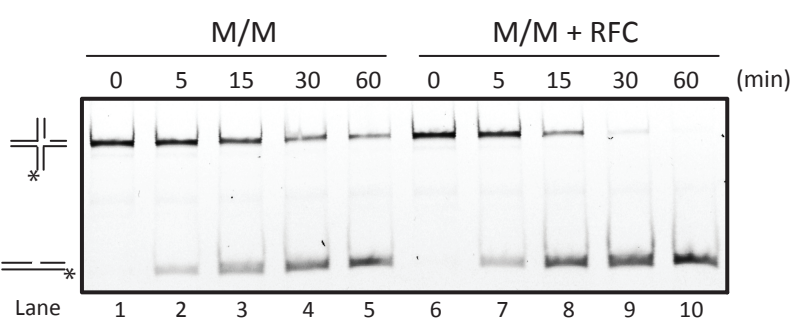

G

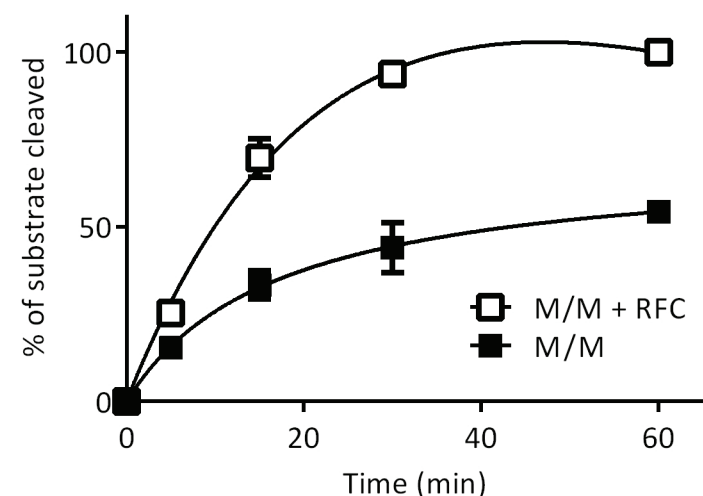

Supplement: Supplementary file 3 — Stimulation of Mus81-Mms4 nuclease activity by RFC complex. (A) RFC stimulation of human MUS81-EME1 complex. Reaction mixtures containing DNA substrate (5 nM) and the Mus81-Mms4 (0.25 nM, lanes 1–5) or MUS81-EME1 (0.25 nM, lanes 6–10) were incubated with increasing amounts of RFC complex (0.25, 0.5, 1.25, and 2.5 nM) at 37 °C for 20 min and then analyzed. (B–G) Time course enhancement of the Mus81 complex nuclease activity by RFC on various DNA substrates. Mus81-Mms4 (0.2 nM) was incubated with indicated DNA substrates (4 nM) in the presence or absence of RFC (2 nM). Reactions were incubated at 37 °C for 60 min. Aliquots of the reactions were taken at the indicated times and analyzed. Quantification of data from three independent experiments was performed for each DNA substrate. *Raw data provided in Additional file 2. (PDF 2850 kb) [file 12915_2017_429_MOESM3_ESM.pdf]

### Supplementary Figure 3

**A**

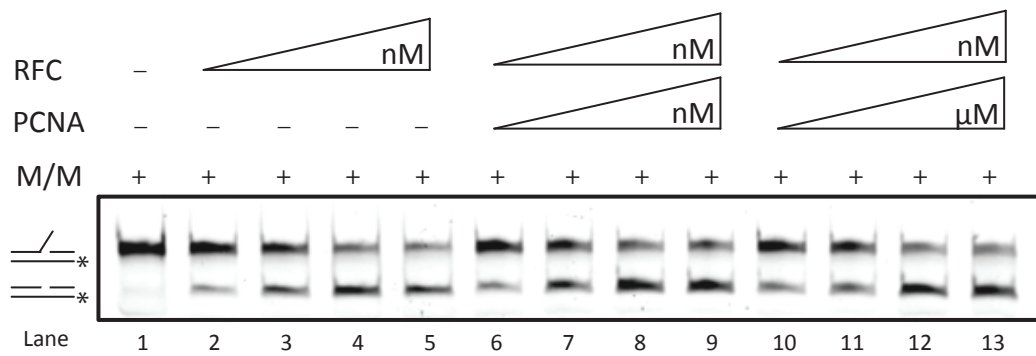

# B

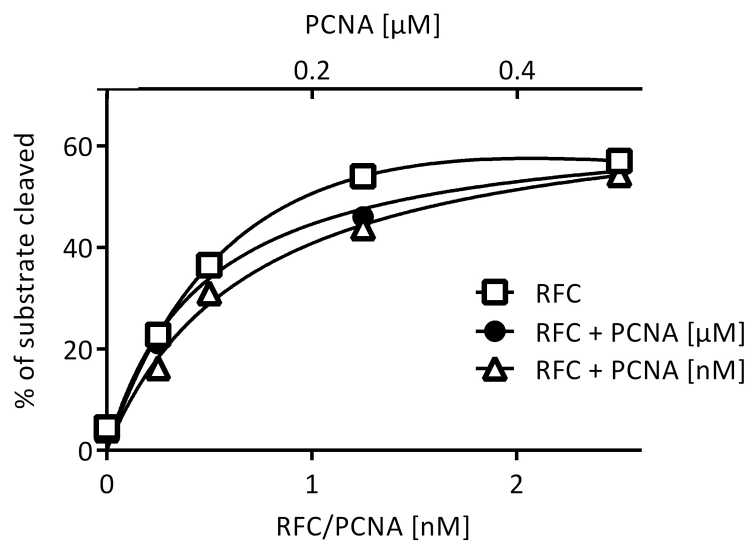

C

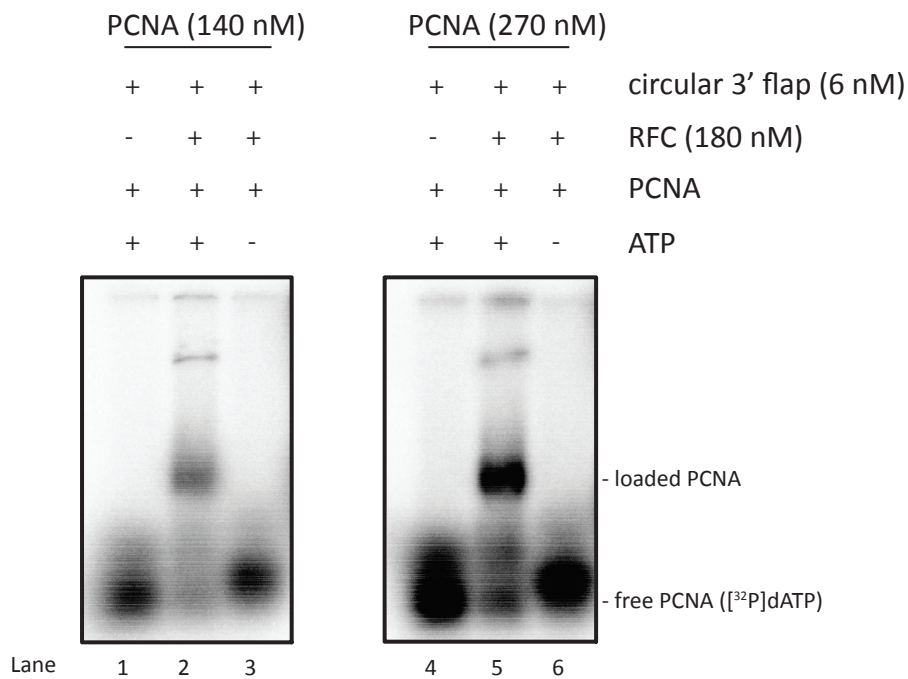

Supplement: Supplementary file 4 — Effect of RFC and PCNA on nuclease activity of the Mus81 complex. (A) Effect of PCNA (0.25, 0.5, 1.25, and 2.5 nM or 0.05, 0.1, 0.25, and 0.5 μM) in the presence of RFC (0.25, 0.5, 1.25, and 2.5 nM) on the nuclease activity of the Mus81 complex. Standard endonuclease assay (37 °C, 20 min) was performed with the indicated amounts of enzyme. (B) Quantification of products formed in panel A. (C) PCNA is efficiently loaded on circular 3′ flap substrate. Loading reaction of 32P-PCNA (140, 270 nM) on circular 3′ flap DNA substrate (6 nM) was performed in the presence or absence of RFC (180 nM) and ATP (1 mM) as indicated. *Raw data provided in Additional file 2. (PDF 1762 kb) [file 12915_2017_429_MOESM4_ESM.pdf]

Supplementary Figure 4

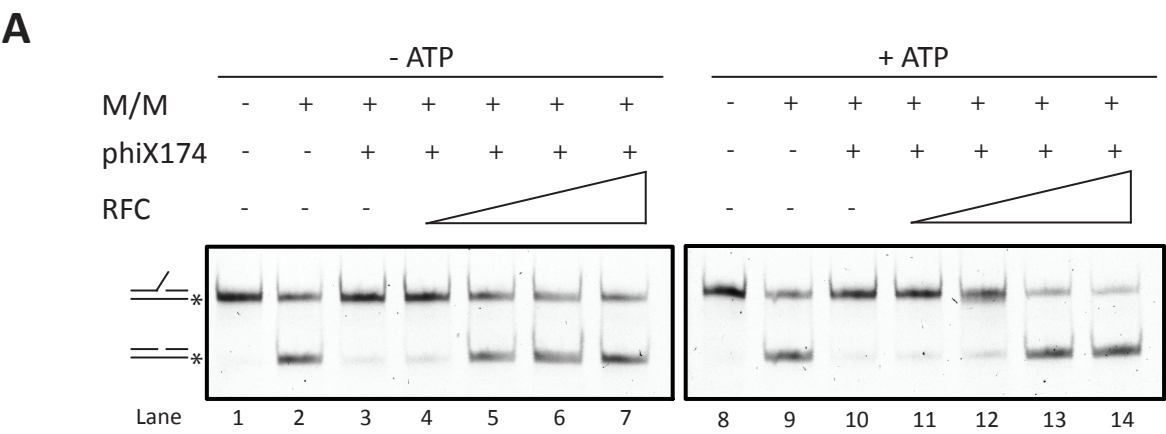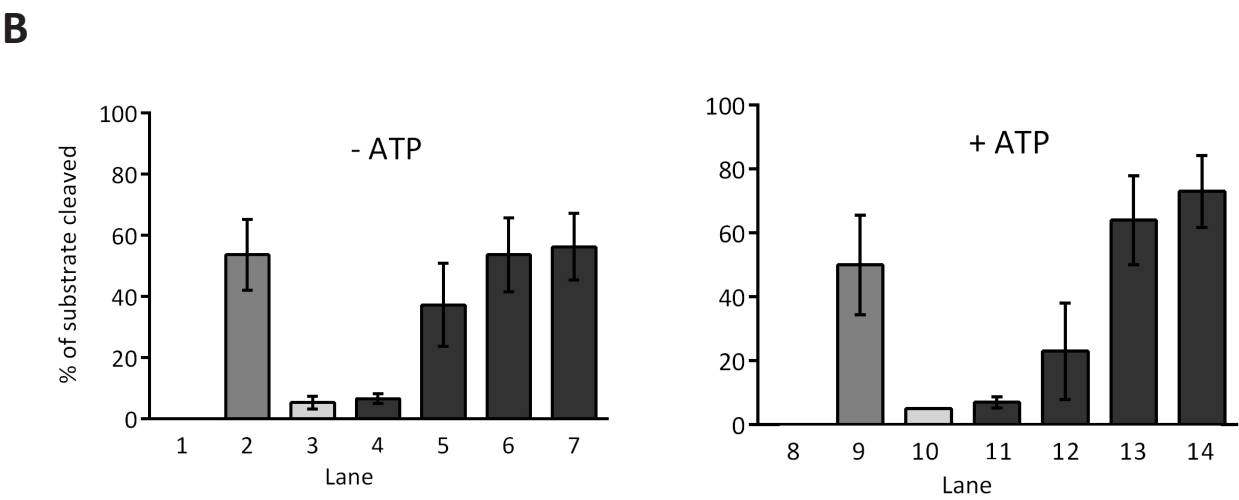

Supplement: Supplementary file 5 — Effect of ATP on substrate targeting of the Mus81 complex by RFC. (A) Mus81-Mms4 (0.4 nM) was incubated with 3′ flap substrate (4 nM) in the presence or absence of ΦX174 virion circular ssDNA (0.25 nM), ATP (1 mM), and increasing concentrations of RFC (5, 12.5, 25, 50 nM). Reactions were incubated at 37 °C for 30 min and analyzed. (B) Quantification of the data in A from three independent experiments. *Raw data provided in Additional file 2. (PDF 1432 kb) [file 12915_2017_429_MOESM5_ESM.pdf]

Supplementary Figure 5

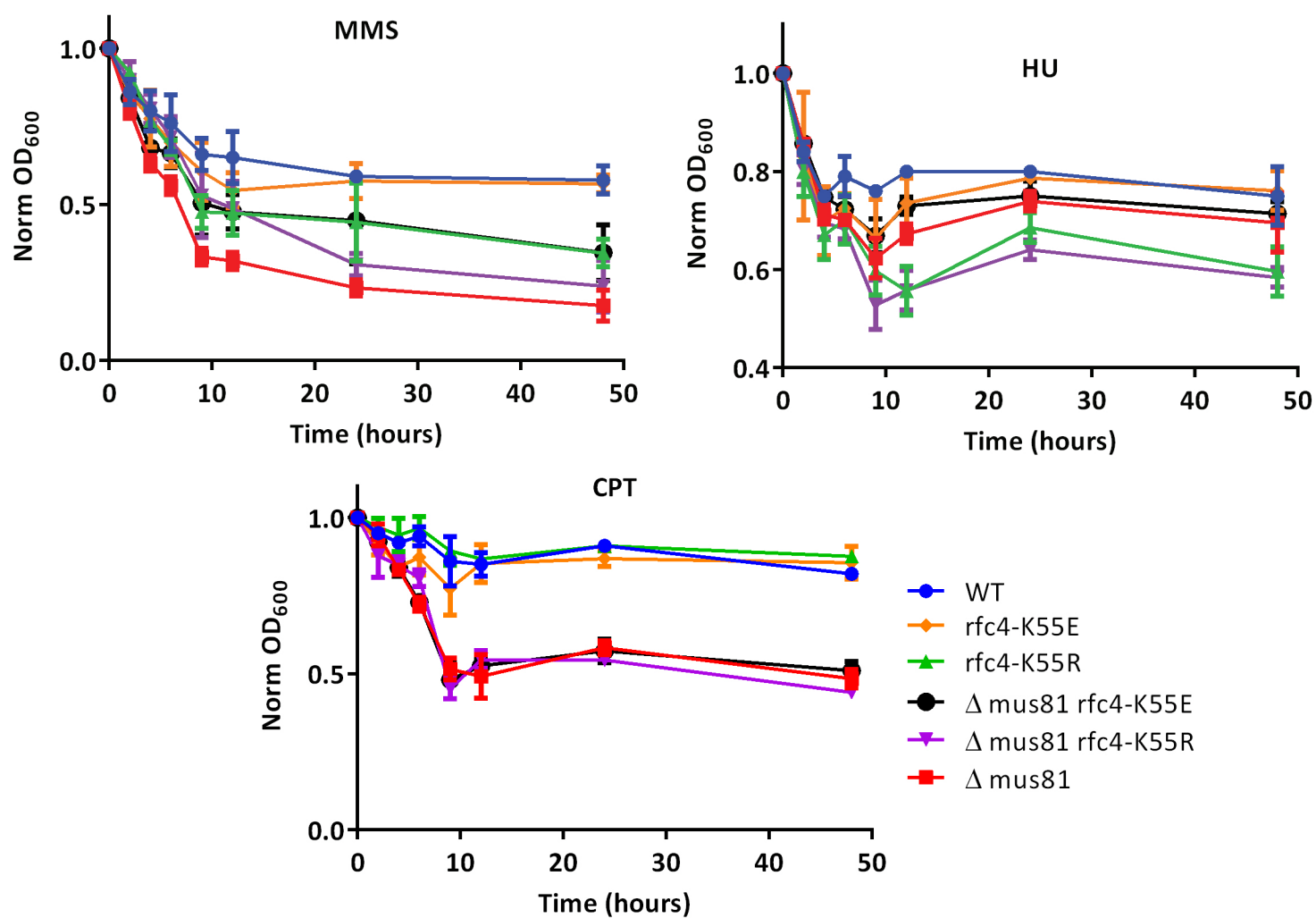

Supplement: Supplementary file 7 — Analysis of cell growth in liquid medium. Yeast cells (biological triplicates for each strain) were treated by various DNA-damaging agents (CPT (5 μg/mL), MMS (0.01%), HU (50 mM)) and the cell growth was analyzed by OD600 measurement at the indicated times. *Raw data provided in Additional file 8. (PDF 932 kb) [file 12915_2017_429_MOESM7_ESM.pdf]
