# Supplementary material for: Role of PCNA and RFC in promoting Mus81-complex activity
Source: BMC Biol. 2017 Oct 2;15:90. doi: 10.1186/s12915-017-0429-8 (PMC5625722; doi:10.1186/s12915-017-0429-8)
Supplement: Supplementary file 9 — (A) Strains and (B) plasmids used in this study. (DOC 36 kb) [file 12915_2017_429_MOESM9_ESM.doc]

**Supplementary Table 1:** **A)** Strains and **B)** plasmids used in this study.

1. Strains

| **Strain** | **Genotype** | **Ref.** |
| --- | --- | --- |
| WT | MAT**α***ade2-1 can1-100 ura3-1 his3-11,15 leu2-3,112 trp1-1* | This study |
| *∆mus81* | MAT**α** *ade2-1 can1-100 ura3-1 leu2-3,112 trp1-1 ΔMUS81::HIS5* | This study |
| *rfc4-K55R* | MAT**α** *ade2-1 his3-11,15 ura3-1 trp1-1 leu2-3,112 can1-100 ΔRFC4∷kanMX6*+ [pBL625] | This study |
| *∆mus81 rfc4-K55R* | MAT**α** *ade2-1 his3-11,15 ura3-1 trp1-1 leu2-3,112 can1-100 ΔRFC4∷kanMX6 ΔMUS81::HIS5* + [pBL625] | This study |
| *rfc4-K55E* | MAT**α** *ade2-1 his3-11,15 ura3-1 trp1-1 leu2-3,112 can1-100 ΔRFC4∷kanMX6*+ [pBL633-E] | This study |
| *∆mus81 rfc4-K55E* | MAT**α** *ade2-1 his3-11,15 ura3-1 trp1-1 leu2-3,112 can1-100 ΔRFC4∷kanMX6 ΔMUS81::HIS5*+ [pBL633-E] | This study |
| PY94 | MAT**a***,* ade2-1 his3-11,15 ura3-1 trp1-1 leu2-3,112 can1-100 ΔRFC4∷kanMX6 + [pBL619] | Schmidt et al., 2001 |
| RFC2-FLAG | MAT**a** RFC2-FLAG::TRP1 *ade1 his2 trp1 ura3 leu2* | Naiki et al, 2001 |
| RFC4-FLAG | MAT**a** RFC4-FLAG::URA3 *ade1 his2 trp1 ura3 leu2* | Naiki et al, 2001 |

1. Plasmids

| pBL619 | RFC4, URA3, CEN6,ARSH4 | Schmidt et al., 2001 |
| --- | --- | --- |
| pBL633-E | *rfc4–K55E, HIS3, CEN6,ARSH4* | Schmidt et al., 2001 |
| pBL625 | *rfc4–K55R, TRP1,CEN6, ARSH4* | Schmidt et al., 2001 |
