# Supplementary material for: Role of PCNA and RFC in promoting Mus81-complex activity
Source: BMC Biol. 2017 Oct 2;15:90. doi: 10.1186/s12915-017-0429-8 (PMC5625722; doi:10.1186/s12915-017-0429-8)

**Supplementary Table 2:** Oligonucleotides used in this study together with the structures of DNA substrates. Fluorescence is marked by asterisk.

| **Oligonucleotide** | **Sequence** |
| --- | --- |
| Oligo 1 | 5´- AGCTACCATGCCTGCACGAATTAAGCAATTCGTAATCATGGTCATAGCT -3´ |
| Oligo 2 | 5´- AGCTATGACCATGATTACGAATTGCTTGGAATCCTGACGAACTGTAG -3´ |
| Oligo 3 | 5´- AATTCGTGCAGGCATGGTAGCT -3´ |
| Oligo 4 | 5´- AGCTATGACCATGATTACGAATTGCTT -3´ |
| Oligo 5 | 5´- GATGTCAAGCAGTCCTAAGGAATTCGTGCAGGCATGGTAGCT -3´ |
| Oligo 6 | 5´- AGCTATGACCATGATTACGAATTGCTTGGAATCCTGACGAACTGTAG -3´ |
| Oligo 7 | 5´- CTACAGTTCGTCAGGATTCC -3´ |
| Oligo 8 | 5´- CAACGTCATAGACGATTACATTGCTACATGGAGCTGTCTAGAGGATCCGA -3´ |
| Oligo 9 | 5´- TGGGTCAACGTGGGCAAAGATGTCCTAGCAATGTAATCGTCTATGACGTT -3´ |
| Oligo 10 | 5´- GTCGGATCCTCTAGACAGCTCCATGATCACTGGCACTGGTAGAATTCGGC -3´ |
| Oligo 11 | 5´- GGACATCTTTGCCCACGTTGACCC -3´ |
| Oligo 12 | 5´- TGCCGAATTCTACCAGTGCCAGTGAT -3´ |
| Oligo 13 | 5´-CAAAACGGCAGAAGCCTGAATGAGCTTAATAGAGGTACGATAGACATGAGCCTAC-3´ |
| Oligo 14 | 5´-CCAAAGCGGTCTGGAAACGTACGGATTGTTCAGTA-3´ |
| Mus81-His-For | 5´-CAAAGTTTCAAAGGATTGATACGAACACACATTCCTAGCATGAAAGCCCAG  ATCTGTTTAGCTTGCC -3´ |
| Mus81-His-Rev | 5´-CTTTTTTCTTTATAAAACCTTGCAGGGATGACTATATTTCAAATTGATGGCG  GCGTTAGTATCG -3´ |
| Mus81VER-For | 5´- GTTCATTATGTCTTTCGTACCTACC -3´ |
| Mus81VER-Rev | 5´- CCATATTAGATATCTGCCTTCCCC -3´ |
| Mus81(1-319) E44Q fwd | 5´- CTGTAGGATAATAAAATGAACCCTGAGCATTTTGTAAATTCCTTTTTGCTTTTTCATAG-3´ |
| Mus81(1-319) E44Q fwd | 5´- CTATGAAAAAGCAAAAAGGAATTTACAAAATGCTCAGGGTTCATTTTATTATCCTACA  G-3´ |


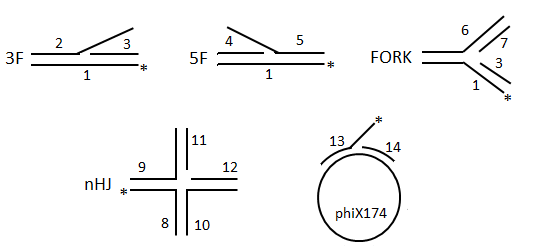

Supplement: Supplementary file 10 — Oligonucleotides used in this study together with the structures of DNA substrates. Fluorescence is marked by an asterisk. (DOC 49 kb) [file 12915_2017_429_MOESM10_ESM.doc]
